# Abnormal Chloride Homeostasis in the Substancia Nigra Pars Reticulata Contributes to Locomotor Deficiency in a Model of Acute Liver Injury

**DOI:** 10.1371/journal.pone.0065194

**Published:** 2013-05-31

**Authors:** Yan-Ling Yang, Jun-Jie Li, Ru Ji, Yan-Yan Wei, Jing Chen, Ke-Feng Dou, Ya-Yun Wang

**Affiliations:** 1 Department of Anatomy and K.K. Leung Brain Research Centre, The Fourth Military Medical University, Xi'an, China; 2 Department of Hepatobiliary Surgery, Xi-Jing Hospital, The Fourth Military Medical University, Xi'an, China; Baylor College of Medicine, Jiao Tong University School of Medicine, United States of America

## Abstract

**Background:**

Altered chloride homeostasis has been thought to be a risk factor for several brain disorders, while less attention has been paid to its role in liver disease. We aimed to analyze the involvement and possible mechanisms of altered chloride homeostasis of GABAergic neurons within the substantia nigra pars reticulata (SNr) in the motor deficit observed in a model of encephalopathy caused by acute liver failure, by using glutamic acid decarboxylase 67 - green fluorescent protein knock-in transgenic mice.

**Methods:**

Alterations in intracellular chloride concentration in GABAergic neurons within the SNr and changes in the expression of two dominant chloride homeostasis-regulating genes, KCC2 and NKCC1, were evaluated in mice with hypolocomotion due to hepatic encephalopathy (HE). The effects of pharmacological blockade and/or activation of KCC2 and NKCC1 functions with their specific inhibitors and/or activators on the motor activity were assessed.

**Results:**

In our mouse model of acute liver injury, chloride imaging indicated an increase in local intracellular chloride concentration in SNr GABAergic neurons. In addition, the mRNA and protein levels of KCC2 were reduced, particularly on neuronal cell membranes; in contrast, NKCC1 expression remained unaffected. Furthermore, blockage of KCC2 reduced motor activity in the normal mice and led to a further deteriorated hypolocomotion in HE mice. Blockade of NKCC1 was not able to normalize motor activity in mice with liver failure.

**Conclusion:**

Our data suggest that altered chloride homeostasis is likely involved in the pathophysiology of hypolocomotion following HE. Drugs aimed at restoring normal chloride homeostasis would be a potential treatment for hepatic failure.

## Introduction

Hepatic encephalopathy (HE) is a major neuropsychiatric disorder that occurs in patients with severe liver failure [1]. Patients with the acute form of HE show altered motor function and loss of coordination, including psychomotor slowing, bradykinesia, hypokinesia, tremor, or gait impairment, which reduces their quality of life [2]. The mechanisms by which liver failure leads to altered motor function remained unclear. These motor alterations are reproduced in animals receiving the toxin thioacetamide (TAA) [3]. TAA is a hepatotoxic and hepatocarcinogenic agent that is largely used as an inducer of acute HE in animal model studies [4], [5].

The motor symptoms of HE have been attributed to dysfunction of the basal ganglia [6] or alterations of the neuronal circuits between the basal ganglia and the prefrontal cortex [7], [8]. Magnetic resonance images and positron emission tomography results from clinical patients with liver diseases have supported the role of basal ganglia in HE [9]–[11]. Substantia nigra pars reticulata (SNr) is a mesencephalic nucleus that functions as a relay area for basal ganglia output [12]. This structure is composed of GABA-containing projection neurons that receive GABA-mediated input from the striatum and globus pallidus and project their axons to thalamic motor nuclei, superior colliculus and brainstem motor areas as well as to dopamine neurons of the SN pars compacta [12]. Pharmacological evidence indicates that GABA in SNr is involved in various aspects of motor function [13]–[17]. However, much less is known about the role of SNr GABA in HE.

The Na^+^-K^+^-2Cl^−^ co-transporter (NKCC1, Cl^−^-uptake) and the K^+^-Cl^−^ co-transporter (KCC2, Cl^−^-extrusion) are the most important known chloride regulators in brain [18], [19]. Alterations in the balance of NKCC1 and KCC2 may determine the switch from a hyperpolarizing to a depolarizing effect of GABA [18], [19]. Recent studies have highlighted that abnormal chloride homeostasis in different areas of the central nervous system is associated with brain disorders, including epilepsy, neuropathic pain, brain injury and axotomy [20]–[25]. The finding that chloride homeostasis is altered in certain neurological disorders forces an assessment of the degree of chloride accumulation in cells to guide treatment involving GABA-modulating drugs. However, no clear indication of abnormal chloride homeostasis has been shown in HE.

In the present study, glutamic acid decarboxylase 67 (GAD67) - green fluorescent protein (GFP) knock-in transgenic mice (C57BL/6 mice strain) were used to identify GABAergic neurons within the SNr [26]. We assessed intracellular chloride concentration ([Cl^−^]_i_) in GABAergic neurons within the SNr and the expression of the two dominant chloride homeostasis-regulating genes (KCC2 and NKCC1) within this area in mice with hypolocomotion induced by TAA. Moreover, the effects of pharmacological blockade and/or induction of KCC2 and NKCC1 functions with specific inhibitors/activators in the locomotor activity were assessed. Investigations herein performed include the analyses of: (a) locomotor activity by open field test, (b) liver injury by histology staining and ELISA, (c) [Cl^−^]i by fluorometric measurement, (d) changes in KCC2 and NKCC1 mRNA and protein expression levels by real-time quantitative RT-PCR and Western blot analysis, (e) neuronal counting after morphological staining, (f) blood and brain ammonia determination by ELISA, (g) brain water content evaluation by gravimetric method, and (h) neuropharmacological evaluation after intranigral or systemic administration of drugs. In order to address whether or not the mouse genetic model would affect the measured parameters, a series of parallel experiments have been performed on C57BL/6 mice and analysed. Overall, our results are consistent with the involvement of a downregulation in KCC2 chloride channel expression levels within SNr GABAergic neurons mediating the hypolocomotion phenotype in an acute liver injury model.

## Materials and Methods

### Animals and experimental design

This research was approved by the Institutional Animal Use and Care Committee of The Fourth Military Medical University (China), and this protocol complied with the Guide for the Care and Use of Laboratory Animal (NIH Publication, 1996). Adult male GAD67 - GFP knock-in C57BL/6 mice and wild type C57BL/6 mice (called GAD67 – GFP mice and wild type mice, respectively) weighing 25–35 g were used in this study. GAD67-GFP knock-in mice used were generously provided by Dr. Tamamaki N (Department of Morphological Brain Science, Graduate School of Medicine, Kyoto University, Japan) and bred in our animal facility; the generation of these mice has been described by Tamamaki et al. [26]. The animals were maintained in a 12 h light/dark cycle with free access to standard laboratory feed and water.

We have first characterize the model, after 3 days of TAA treatment. Acute liver injury and hypolocomotor phenotypes were assessed by serum detection of hepatic enzymes and histological staining of liver and locomotor evaluations and an open field test, respectively. Mice showing a reduced locomotor activity were used in subsequent examinations, including measurements of intracellular chloride concentration in SNr GABAergic neurons as well as associated changes in two dominant chloride homeostasis-regulating genes (KCC2 and NKCC1) at both mRNA and protein levels. To assess whether TAA might alter the number of GABAergic neurons in the SNr, Nissl staining was combined with double immunostaining with an anti - GFP antibody to detect GABAergic neurons located in the SNr and an anti - tyrosine hydroxylase (TH) antibody to detect dopaminergic neurons located in the substantia nigra pars compacta (SNc). We have then analyzed the effects of the unilateral pharmacological blockade of KCC2 and NKCC1 function with their specific inhibitors on motor activity were investigated.

### Induction of acute HE

A well-characterized model of TAA-induced acute HE was used in the study [3]–[5]. Briefly, HE was induced by daily intraperitoneal (i.p.) injections of TAA (Sigma, St. Louis, MO, USA) (485 mg/kg, dissolved in 0.3 ml 0.9% NaCl) on 3 consecutive days. Sham controls received 0.3 ml of saline (i.p.). Supportive therapy consisting of 25 ml/kg 0.45% NaCl, 5% dextrose and 20 mM KCl was administered subcutaneously every 12 h after the first injection of TAA, with the aim of preventing renal failure, hypoglycemia and electrolyte imbalance [4]. Similar therapy was applied to sham controls. After experimental treatments, mice were intermittently exposed to an infrared lamp to maintain body temperature.

### Assessment of neurological function

Neurological function was assessed by a 10-point scale based on reflexes and task performance: exit from a circle 1 m in diameter in less than 1 min, seeking, walking a straight line, startle reflex, grasping reflex, righting reflex, placing reflex, corneal reflex, maintaining balance on a beam of 3, 2 and 1 cm in width, climbing onto a square and a round pole [27]. A score of 1 was assigned for each task failed or for an abnormal reflex reaction. The pathological scores correlate very well with clinical disability scores and with the degree of HE [28]. Failure to perform task was scored as one point; and a success as zero points. Hence, animals with normal behavior would receive a score of 0, reflecting healthy behavior, whereas a score of 10 would reflect maximal neurological impairment. The following measures were particularly sensitive to TAA: walking a straight line, maintaining balance on a beam 3, 2 and 1 cm in width and climbing onto a square and a round pole. A hypolocomotor phenotype was considered to be developed when animals were showing neurological scores between 4 an 7. The analyses were performed by an investigator who was blinded to treatment.

### Histological analysis

Livers were removed immediately after cervical dislocation and fixed in 10% buffered formalin. Paraffin sections (5.0 µm) were stained with hematoxylin and eosin (H & E) and analyzed using an Olympus BX51 microscope. Six liver sections from each animal were used for analysis. Liver damage was also evaluated by serum levels of aspartate aminotransferase (AST), alanine aminotransferase (ALT) and lactate dehydrogenase (LDH) using standard kits (Tiangen, Shanghai, China).

### Locomotor activity assessment by open field test

The locomotor responses of GAD67-GFP and wild type mice were measured with individual locomotor activity boxes (9×20×11 cm; Imetronic), as previously described [29]. Mice were habituated to the locomotor cages daily during 10 min on three consecutive days. In brief, mice were placed in the locomotor cages, and horizontal and vertical locomotor activity was recorded for 10 min. The light inside the apparatus was maintained at a minimum level to avoid anxiety behavior. After each test the apparatus was cleaned with a solution of 10% ethanol for removal of odors from other animals.

### Tissue preparation and fluorometric measurement of [Cl^−^]_i_

Tissue preparation and fluorometric measurement of [Cl^−^]_i_ were performed as previously described with minor modifications [30]. Single SNr neurons were acutely isolated by enzymatic digestion and mechanical dispersion from control and TAA - treated GAD67 - GFP mice. Briefly, 400–500 µm thick vibratome cut brain slices containing the substantia nigra were incubated for 2 h at 32°C in modified Krebs' solution (in mM: NaCl 128, KCl 5, CaCl_2_ 2.7, MgSO_4_ 1.2, Na_2_HPO_4_ 1, glucose 16, N – 2 – hydroxyethylpiperazine - N0 - 2 - ethanesulfonic acid (HEPES) 10, pH adjusted to 7.4 by NaOH), bubbled with 5% CO_2_ - 95% O_2_ and then placed into modified Krebs' solution containing 1.5 mg/ml protease at 32°C for 40 min. The slices were then transferred into a chamber, and the SNr was identified under a fluorescence microscope (Keyence, Japan) and carefully dissected with a razor blade. Brain fragments collected from several brain slices were treated by gentle pipetting using a fine glass tube (inner tip diameter, 150 µm). The dissociated cells were observed under a fluorescence microscope (CKX41, Olympus, Japan), and GFP - positive cells were identified in photographs taken with a digital CCD camera (MP3.3 - RTV, Qimaging, Canada). The isolated GABAergic neurons were kept in the extracellular solution at room temperature (20–22°C). These neurons remained visible for fluorometric measurement of [Cl^−^]_i_ for up to 4–5 h.

The cells were then incubated for 1 h with a Cl^−^ - sensitive fluorescent dye, 10 mM N - (6-methoxyquinolyl) acetoethyl ester (MQAE) (Dojindo, Japan) [30], rinsed and perfused with modified Krebs' solution. Measurement of the fluorescence intensity of a single SNr neuron was recorded using a confocal microscope (FV - 1000, Olympus, Japan) with excitation and emission wavelengths of 360 and 535 nm, respectively. The relationship between the MQAE fluorescence and the intracellular concentration of chloride was previously found to be expressed by the linear function 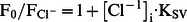 [30]. F_0_ is the background fluorescence obtained by quenching the MQAE signal with a HEPES-buffered KSCN (150 mM, pH 7.2) solution at the end of the experiments. 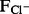 is the fluorescence level of the selected neurons in the experiment. K_SV_ is the Stern-Volmer constant for collisional quenching. For determination of K_SV_, the MQAE signal should be calibrated against [Cl^−^]_i_ by exposing the cells to a K - rich HEPES buffer (pH 7.0) containing various Cl^−^ concentrations with NO_3_^−^ as the substituting anion. However, in the present study, we did not determine K_SV_ for the SNr neurons of control and TAA-treated mice because the 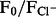 was used as a parameter that is proportional to the Cl^−^ concentration. Therefore, we only obtained information about differences in [Cl^−^]_i_, but did not determine the absolute [Cl^−^]. SNr neurons that were not loaded with MQAE did not produce background signals with this method. Images were obtained by scanning the excitation light focus through tissue layers 1–3 cells below the dura mater. Mean values of 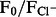 are given with standard deviations (± SD).

### Tissue preparation for real-time quantitative RT-PCR and Western blot analysis

Real-time quantitative RT-PCR and Western blot analysis were performed to analyze changes in mRNA and protein levels of KCC2 and NKCC1 in the SNr of GAD67-GFP and wild type mice after experimental treatment. In brief, mice were anesthetized by ether inhalation, decapitated, and the brain was quickly removed and rinsed with cold, oxygenated Krebs solution. The brain was blocked with a razor blade, fixed using cyanoacrylate glue to the stage of a Vibratome tissue slicer and parasagittal slices (500 µm thick) through the SNr were obtained. These slices were placed in oxygenated Krebs solution at room temperature. It was possible to delineate the SNr with a stereomicroscope using transmitted light.

### Real-time quantitative RT-PCR

Total RNA within the SNr was extracted with Trizol (Gibco BRL, USA). Single stranded complementary DNA (cDNA) was synthesized with oligo (dT)_12–18_ using Superscript™ III Reverse Transcriptase for RT-PCR (Invitrogen, Carlsbad, CA). GAPDH served as an endogenous internal standard control. Equal amounts of RNA (1 µg) were used to prepare cDNA using the SYBR Premix Ex Taq™ (Takara, RR041A) and analyzed by real-time PCR in a detection system (Applied Biosystems). The primer sequences for KCC2, NKCC1 and GAPDH were as follows: KCC2: forward primer, 5-GCGGGATGCCCAGAAGTCTA-3; reverse primer, 5-GATGCAGGCTCCAAACAGAACA-3. Amplification product: 147 bp. NKCC1: forward primer, 5-GTCTCCATGAAAGCTGCAAA-3; reverse primer, 5- AGTCGAACTTGGCGGTAACT-3. Amplification product: 121 bp. GAPDH: forward primer, 5-TGTGTCCGTCGTGGATCTGA-3; reverse primer: 5-TTGCTGTTGAAGTCGCAGGAG-3. Amplification product: 150 bp. The amplification protocol was 3 min at 95°C, followed by 45 cycles of 10 s at 95°C for denaturation and 45 s at 60°C for annealing and extension. All experiments were repeated twice and, in each experiment, PCR reactions were done in triplicate. Target DNA sequence quantities were estimated from the threshold amplification cycle number (Ct) using Sequence Detection System software (Applied Biosystems). The PCR products were analyzed on a 2% agarose gel and visualized with ethidium bromide.

### Western blot analysis

Portions of the SNr were collected into ice-cold homogenization buffer (50 mM Tris - HCl, pH 7.4, 150 mM NaCl, 1 mM EDTA, 0.5% Triton X, protease inhibitor cocktail) and homogenized before centrifugation (14,000 g). Aliquots of the supernatant were incubated for 30 min at 37°C in Laemmli SDS sample buffer (Invitrogen, Carlsbad, CA, USA). 10 to 20 µg of total protein was separated on 4–12% SDS – PAGE Bis – Tris gels (Novex, Invitrogen, Carlsbad, CA, USA) and immunoblotted on nitrocellulose. Membranes were incubated with anti - KCC2 (1/1000, Upstate, Temecula, CA, USA), anti-SLC12A2 (NKCC1) antibody (1/1000, Sigma), or anti-actin antibodies (1/2000, Sigma), followed by incubation with horseradish peroxidase - linked anti - rabbit secondary antibody (1/10,000, Santa Cruz Biotechnology, Santa Cruz, CA, USA) or anti-mouse secondary antibody (1/10,000, Santa Cruz Biotechnology). Immunoblots were developed using an ECL Western-blotting protocol (Enhanced Chemiluminescence, Lumilight Roche Applied Science, Indianapolis, IN, USA). For sequential analysis of Western blot membranes, previously bound antibodies were removed with stripping buffer (Pierce, Rockford, Il, USA). Quantification of immunoreactivity was performed with densitometric scanning using Quantity One software (BioRad, San Diego, CA, USA). For each animal, band intensities were normalized by calculating the ratio of the intensity of bands corresponding to KCC2 to the intensity of the band corresponding to actin. The actin-normalized data for each lane were then expressed as a percentage of the group mean of values obtained from all control mice run on the same gel.

### Double immunofluorescence labeling

GAD67-GFP and wild type mice were deeply anesthetized with chloral hydrate (5 mg/100 g body weight) and perfused transcardially with 0.1 M phosphate buffer (pH 7.4) containing 4% paraformaldehyde. The brains were removed, postfixed with the same fixative for 4 h, then placed in 0.05 M diethylpyrocarbonate-treated phosphate buffered saline (PBS; pH 7.4) containing 30% sucrose (w/v) overnight at 4°C. Sagittal sections (40 µm) throughout the entire SNr were cut on a cryostat. The sections, which were fixed as described above, were incubated overnight in PBS with a mixture of 1 µg/ml guinea pig anti - GFP antibody (Sigma) and one of the following antibodies: rabbit anti - KCC2 serum (1/500, Santa Cruz, CA, USA), or rabbit Anti-SLC12A2 (NKCC1) antibody (1/500, Sigma), or mouse anti - TH IgG (1/200, BD Biosciences Pharmingen, San Diego, CA, USA). After a rinse with PBS, the sections were incubated for 3 h in PBS with 4 µg/ml Alexa 488 - conjugated goat anti - guinea pig IgG antibody and 4 µg/ml Alexa 594 - conjugated donkey anti - goat or anti - mouse IgG (Molecular Probes, USA) in the presence of 10% (v/v) normal rabbit or mouse serum, respectively. The sections were mounted onto gelatinized glass slides and coverslipped with 50% (v/v) glycerol and 2.5% (w/v) triethylenediamine (anti - fading reagent) in PBS. The sections were observed under a confocal microscope (FV - 1000, Olympus, Japan) with a confocal depth of 1.0 µm, appropriate laser beams and filters for Alexa 488 (excitation: 488 nm; emission: 505–530 nm) and Alexa 594 (excitation: 543 nm; emission: 560 nm). A 10× objective lens (10×/numerical aperture 0.3) and a 40× oil-immersion objective lens (40×/numerical aperture 1.3 oil) were used. A pinhole size of 67 µm resulted in optical sections of <10 µm thickness (10× objective lens) and <1 µm (40× objective lens). Finally we performed control experiments in which the primary or secondary antibody was omitted. No labeling was observed under these conditions.

### Subcellular detection of KCC2 - immunolabeling on GABAergic neurons within the SNr

As described in a previous report [31], KCC2 immunolabeling of plasma membranes in GABAergic neurons was quantified. Briefly, Zeiss software (version 4) was used to overlay the internal and external borders of the KCC2 - immunolabeled membrane on serial sections of substantia nigra. The delineated area was extracted and imported the digital image using the Image J software (version 1.38 x, National Institute of Health, USA). The ratios of labeled pixel areas per somatic perimeters were calculated to give an index of the mean density of the immunolabeling within the membrane. Cytoplasmic KCC2 - immunolabeled clusters were quantified in mice of different groups from images digitized under higher laser intensity. Ratios of cluster numbers per somatic area were calculated and compared. The primary antibody by a normal rabbit immunoglobulin fraction (same concentration) was replaced to be a negative control, which was observed as a complete absence of labeling. The specificity of the antibody to KCC2 was confirmed by the lower intensity of the signal in mice.

### Nissl staining

GAD67 - GFP and wild type mice with HE and as controls were perfused through the left cardiac ventricle with 10 ml of 10 mM phosphate buffer (pH 7.4) followed by 40 ml of a cold fixative (4% paraformaldehyde in 100 mM phosphate buffer). After perfusion, the brain tissues were quickly removed, postfixed for 18 h with the same fixative at 4°C, and transferred to 10%, then 20%, and then 30% sucrose solution. Sections (40 µm thick) were prepared using a vibratome (Leica, Wetzlar, Germany). Sections were mounted on microscope slides (Fisher, Fair Lawn, NJ, USA) and air - dried. The slides were soaked in cresyl violet working solution (0.02% buffer solution, 0.2% sodium acetate, 0.3% acetic acid) for 7 min, dehydrated with alcohol and xylene, and then mounted. The images were analyzed using an Olympus BX 51 microscope equipped with a DP71 camera and DP – B software (Olympus). Cell counts were performed without knowledge of the experimental conditions. Nissl - positive neuronal cell numbers were manually and blind - test counted within the SNr region. Total cell counts were averaged from at least three sections per animal in the SNr region with equal level. The data were expressed as percent of number for normal control mice.

### Stereotaxic surgery and unilateral intranigral administration of drugs

Surgeries and drug infusions in GAD67 - GFP and wild type mice were performed in accordance with previously developed models [32]. In brief, needle tip placement coordinates corresponded to those in the atlas of Slotnick and Leonard [33] as follows: anteroposterior, 0.6; lateral, 2; dorsoventral, 3.5. unilateral infusion) was made at 0.5 µl/min on the left side. Both blockage and activation of KCC2 were performed by intranigral injection of [(dihydroindenyl)oxy] alkanoic acid (a KCC2 blocker; DIOA, Sigma), and *N*-ethylmaleimide (a KCC2 activator; NEM, Sigma), respectively, in normal GAD67 - GFP mice. Cannulas were implanted just above the left substantia nigra 3–5 d before drug treatment. DIOA was dissolved in 10% DMSO in saline with two concentrations of 10 and 20 µg/0.5 µl per side, as described in previous reports [34]. NEM was dissolved in 10% DMSO in saline with the concentrations of 1 mmol/0.5 µl per side, slightly modified according to previous report [35]. Two hours later, the motor activity of the mice was evaluated.

After behavioral confirmation of hypolocomotion in GAD67 - GFP mice treated with TAA, the cannulas were implanted above the left substantia nigra 3–5 d before TAA treatment. A nigral injection of bumetanide (Sigma, 10 and 20 nmol/0.5 µl on the left side), a selective inhibitor of NKCC1, was then performed, as described in previous reports [34]. Bumetanide was dissolved in artificial cerebrospinal fluid (ACSF) vehicle. The ACSF vehicle was comprised of (in mM) CaCl_2_ ⋅2H_2_O 1.3, KCl 2.6, MgCl_2_ 0.9, NaHCO_3_ 1.0, Na_2_HPO_4_⋅7H_2_O 2.5, NaCl 125, and dextrose 3.5 (pH 7.4). Controls were injected with the same volume of ACSF. Two hours later, the motor activities of the mice were assessed.

All testing was performed blinded to condition and treatment. At the end of the experiment, the animals were killed by an overdose of chloral hydrate, and the brains removed and fixed in 10% of formalin for 24 h. Brain sections (150 µm) were obtained with the vibroslicer. Injection sites were assessed by the location of the cannulae on digitized images of the brain slices. These images were superimposed onto schemes of the brain. Experiments with cannula locations not corresponding to substantia nigra were discarded.

### Blood and brain ammonia estimation

Plasma ammonia level was measured using a commercially available ammonia assay kit, following the manufacturer's protocol (Tiangen). Brain ammonia was measured as previously described [36]. Briefly, at the end of the treatment time, mice were decapitated, brains quickly removed, and cortices from control and experimental animals homogenized. After centrifugation, ammonia levels in the supernatants were measured using a commercially available ammonia assay kit, following the manufacturer's protocol (Tiangen).

### Brain water measurement

The gravimetric method of Marmarou et al. [37] was employed to estimate brain edema. Briefly, at the end of the treatment time, mice were decapitated, brains quickly removed, and cortical tissue (10 mg) was placed in a bromobenzine - kerosine density gradient column. The equilibration points of the brain samples were read at 2 min. Conversion of specific gravity to brain water was performed as previously described [37], [38].

### Statistical analysis

Data are presented as the mean ± standard error of the mean (SEM). One-way repeated-measures ANOVA was used for the analysis of differences between the experimental groups. *P*<0.05 was considered statistically significant.

## Results

### Thioacetamide (TAA) - mediated acute liver failure induces hepatic encephalopathy and hypolocomotion

**Figure 1:**
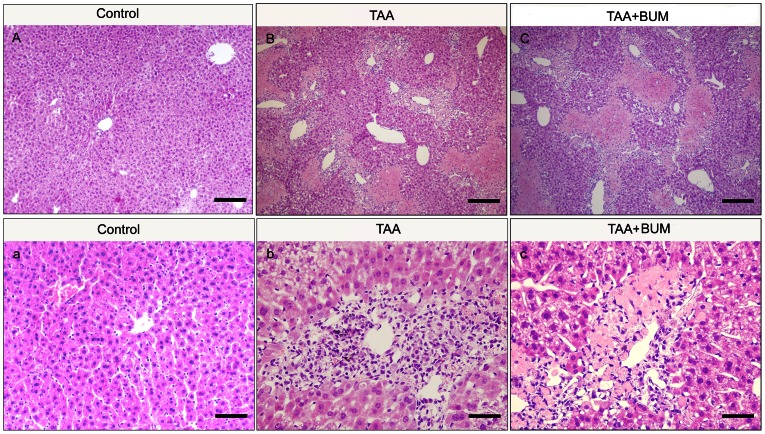
Histology of liver stained with H&E of GAD67-GFP knock-in mice in different groups. Increased necrosis of hepatocytes observed in mice treated with TAA (B and b) compared to normal animals (A and a). Intranigral treatment with bumetanide (BUM) (a selective inhibitor of NKCC1) led to similar liver architecture as that of TAA-injected mice (C and c). a, b and c show a section from A, B and C, respectively, at high magnification. Bars in A, B and C = 30 µm; bars in a, b and c = 3 µm.

As expected from an earlier study [5], after 3 daily i.p. injections of TAA (485 mg/kg), clear signs of hepatic failure with significant increases in ALT (5.8 - fold) and AST (4.6 - fold) (Table 1), together with increased necrosis of hepatocytes were observed in transgenic mice (Figures 1A, a, B and b). In addition, both blood and brain ammonia concentration were significantly increased (*P*<0.05) in TAA-treated animals, when compared to the control group (Table S1).

**Table 1:** Serum levels of aspartate aminotransferase (AST), alanine aminotransferase (ALT) and lactate dehydrogenase (LDH) in different experimental groups.

	AST(U/L)	ALT(U/L)	LDH(U/L)
Control	90.1±4.1	54.7±6.5	37.5±7.0
Sham	88.5±5.0	48.5±4.8	35.8±6.2
TAA	522.3±35.2*	255.3±28.0*	1005.9±60.6*
Control+DIOA	476.7±31.0*	241.2±25.5*	1122.4±63.4*
TAA+BUM	483.9±30.5*	239.9±24.0*	558.0±42.7* ^,^ #

TAA treatment induced clear signs of hepatic failure with significant increases in plasma level of the LDH, accompanied by less dramatic but still significant increases in ALT and AST. The intranigral injection of DIOA (a KCC2 blocker, 20 µg/0.5 µl) did not affect any of the serum transaminases in normal mice. Similarly, the intranigral injection of bumetanide (BUM, a selective inhibitor of NKCC1, a 20 nmol/0.5 µl) could not alter the increases of plasma level of ALT and AST, while ameliorate LDH increase, in mice treated with TAA.

* *p*<0.05 as compared to normal controls;

# *p*<0.05 as compared to TAA group.

We then assessed the development of encephalopathy by measuring neurological scores. All TAA - treated GAD67 - GFP mice showed neurological scores between 4 and 7. Clinical grading of encephalopathy in TAA-treated C57Bl/6 mice was similar to that in TAA-treated GAD67 - GFP mice (data not shown). In addition, brain water was significantly increased (*P*<0.05) in the TAA group when compared to the control group (Table S1). Similar changes in this parameter were observed in wild type mice (data not shown).

**Figure 2:**
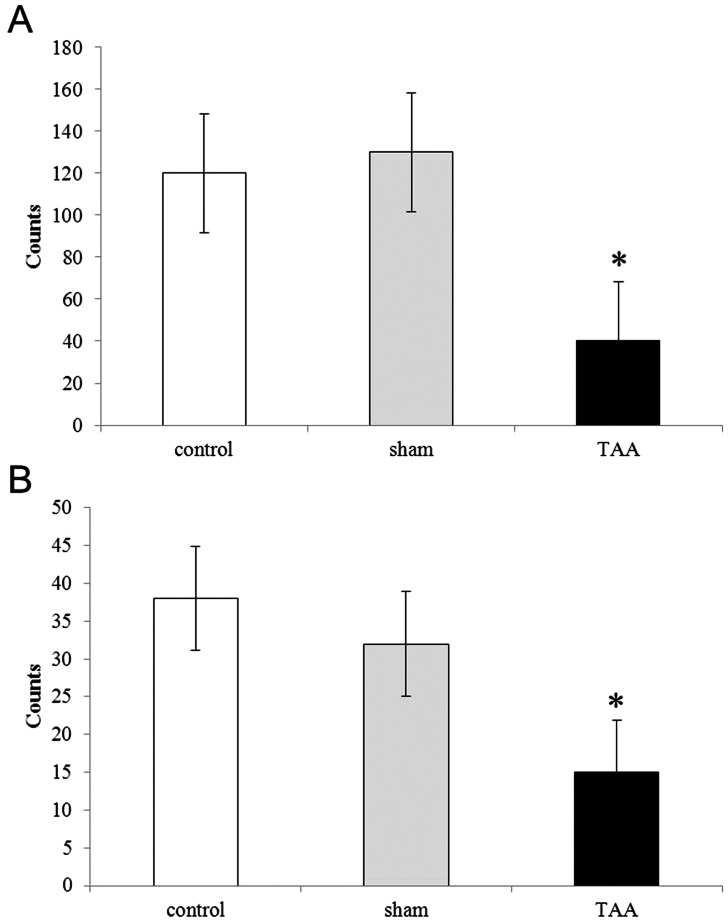
Hypolocomotion induced by TAA in GAD67-GFP knock-in mice, evaluated by open field test. Horizontal (A) and vertical (B) locomotor activity was measured. Data are expressed as the mean ± SEM of photocell counts during a 10 min period. * *P*<0.05 vs normal controls.

Induction of hypolocomotion by TAA treatment was confirmed by open field examinations. As shown in Figure 2, TAA decreased horizontal and vertical locomotor activity in GAD67 - GFP mice, as described before [29]. There were no obvious differences between the normal control group and sham group. Similar findings were observed in wild type mice treated with TAA (not shown). Altogether these results suggest that TAA treatment is able to induce HE and hypolocomotion in both the GAD67-GFP knock in and wild type mice.

### TAA treatment results in increased chloride levels within SNr GABAergic neurons

**Figure 3:**
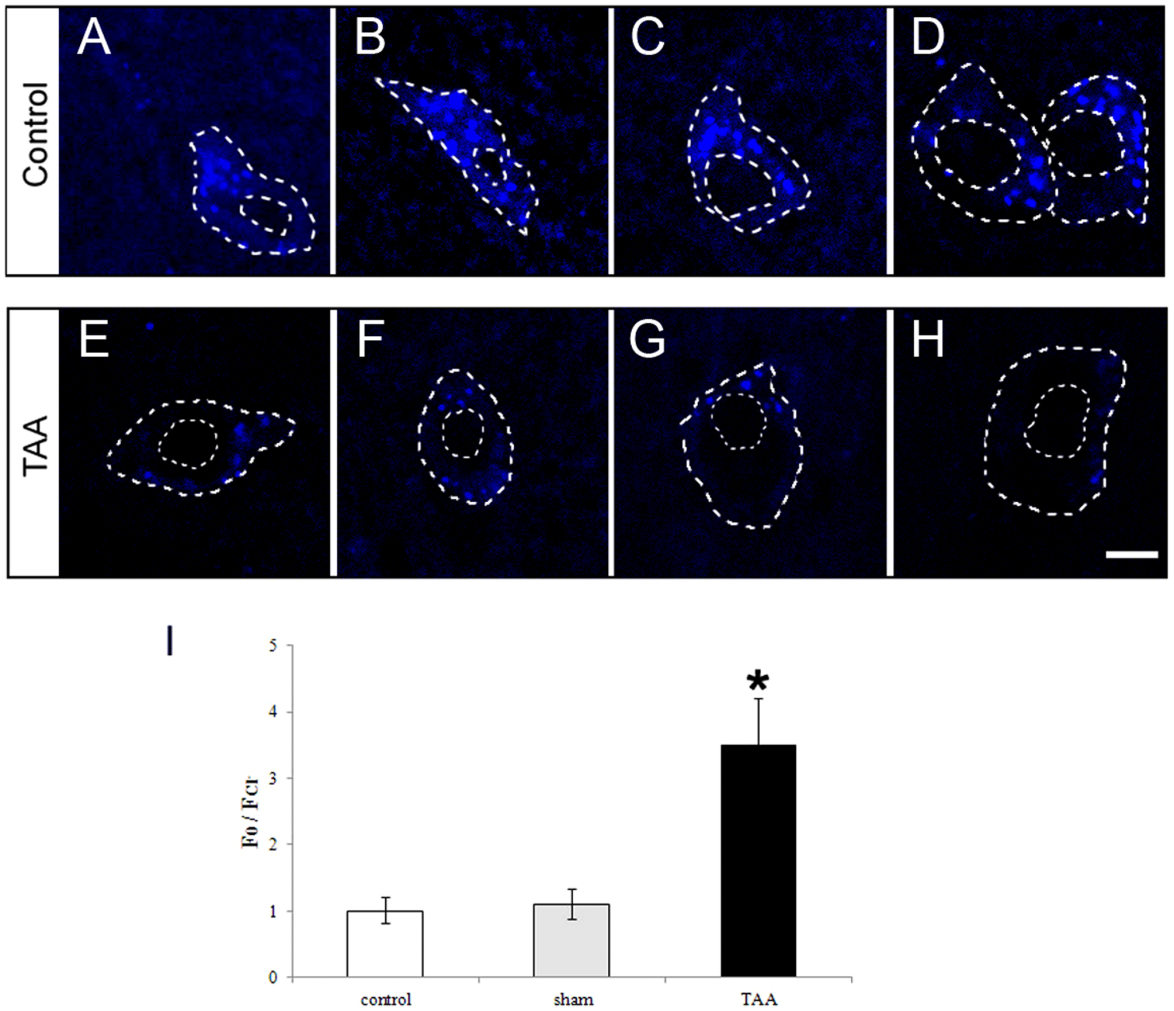
Enhancement of intracellular chloride concentration ([Cl^−^]_i_) following TAA treatment. (A–H) Results of confocal microscopic images of a single neuron within the SNr loaded with the chloride indicator dye MQAE from normal controls (A, B, C and D) and TAA-treated mice (E, F, G and H). The white circles illustrate the area from which the mean fluorescence intensity was obtained. Background subtraction removed most of the pericellular fluorescence as well as the fluorescence originating from the nucleus. All cell sections with intact perimeters were evaluated. The fluorescence is inversely proportional to the Cl^−^ concentration. (I) The corresponding [Cl^−^]_i_ levels were shown as bar graphs. * *P*<0.05 vs normal controls. Bars in A–H = 10 µm.

In order to analyze whether chloride ion levels within GABAergic neurons might change within the SNr, cells were *in vitro* loaded with the Cl^−^ - sensitive fluorescent dye MQAE. The ratio of F0 (the background fluorescence) over 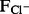 (fluorescence of GABAergic neurons”) was used as a parameter that is directly proportional, according to [Cl^−^]_i_ (see Materials and Methods section). As shown in Figure 3 (A–H), confocal microscope images using specific sensitive dyes showed a decrease in fluorescence intensities within single GABAergic neurons in the SNr in TAA mice, when compared to normal controls. Since application of MQAE results in quenching of the signal directly proportional to intracellular chloride ion levels, these results suggest a significant increase in [Cl^−^]_i_ following TAA treatment (Figure 3I). There was no significant difference in intracellular chloride concentration between untreated and vehicle-treated GAD67-GFP control groups. Similar findings were observed in C57Bl/6 mice treated with TAA (data not shown).

### TAA induces a reduction in KCC2 expression within the SNr and its targeting to cell membrane of GABAergic neurons

**Figure 4:**
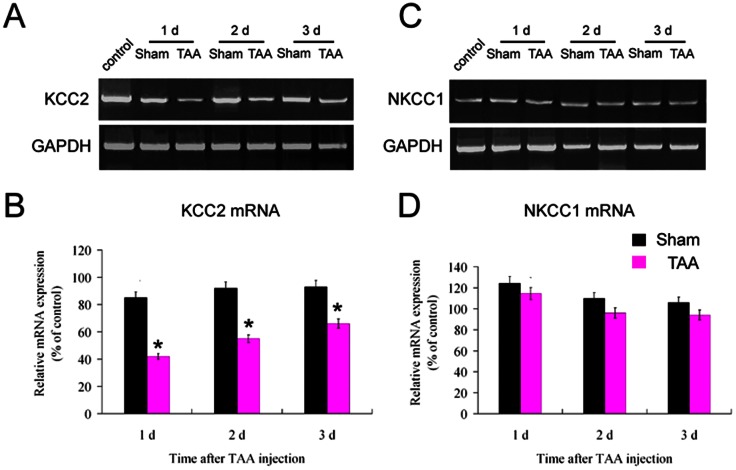
Reduced expression of KCC2 mRNA within the SNr following TAA injection. Real-time quantitative RT-PCR experiments were performed using SYBR green for analysis of KCC2 (A and B) and NKCC1 (C and D) mRNAs expression in SNr of TAA-injected mice at different time points and sham controls. All data were normalized for levels of GAPDH expression within the same sample. Data are calculated as percentages of the average value of controls (B and D). Note that a dramatic down-regulation of KCC2 mRNA was seen at 1 d and maintained up to 3 d in TAA group (B). However, a significant increase in NKCC1 mRNA was observed at 1 d in both the sham and TAA groups, which disappeared at 2 d and 3 d after treatment (D). * *P*<0.05 vs sham controls.

The increase in intracellular chloride levels after TAA treatment suggests the involvement of the expression and/or activity of chloride channels in HE. Thus real-time RT-PCR was used to determine the relative mRNA levels of the two most important chloride channels expressed in the brain [18], [19], KCC2 and NKCCl, in the SNr of GAD67-GFP mice after TAA injection. KCC2 and NKCC1 transcript levels were normalized to levels of GAPDH. As shown in Figure 4A–B, a dramatic down-regulation in KCC2 mRNA levels was observed at 1 d (42.5±4.5% of control, *P*<0.05) and maintained for up to 3 days (66.8±6.2% of control, *P*<0.05) after the last TAA injection, compared to vehicle-treated controls. The expression of KCC2 mRNA at 1 day after vehicle treatment also decreased (85.7±5.0% of control, *P*<0.05); however, KCC2 levels in the SNr of vehicle-treated mice at 2 and 3 days were not significantly different from those of untreated controls (Figure 4A–B, *P*>0.05). As demonstrated in Figure 4D, 1 day after TAA treatment, NKCC1 mRNA levels exhibited a marked increase both in the sham controls (122.5±2.6% of control, *P*<0.05) and the TAA group (114.0±2.8% of control, *P*<0.05). However, there were no significant differences in the expression of NKCC1 between TAA and sham treatments at either 2 or 3 days (*P*>0.05). The level of KCC2 and NKCC1 in wild type mice did not differ from those in GAD67-GFP mice (data not shown).

**Figure 5:**
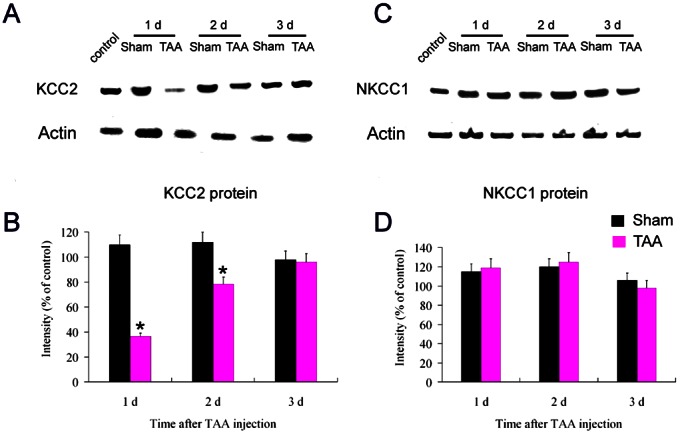
Reduced level of KCC2 protein and unaltered level of NKCC1 protein in the SNr by TAA injection. Western blot experiments were performed for analysis of KCC2 (A and B) and NKCC1 (C and D) proteins in the SNr of TAA-injected and sham mice at different time points. All data have been normalized to actin levels within the same sample. Data are calculated as percentages of the average value of control mice (B and D). Note that a dramatic down-regulation of KCC2 protein was seen at 1 d and 2 d in the TAA group (B). No significant change of NKCC1 protein was seen at different time points in TAA group compared with those in sham group (D). * *P*<0.05 vs sham controls.

Western blotting was employed to determine KCC2 and NKCC1 protein levels in the SNr after the TAA injection. KCC2 and NKCC1 transcript levels were normalized to levels of actin. As shown in Figure 5A and B, a significant decrease in KCC2 expression levels was observed in the SNr at 1 day (36.5±3.0% of control, *P*<0.05) and at 2 days (78.4±4.0% of control, *P*<0.05) in GAD67-GFP mice after the last TAA injection compared to the vehicle-treated controls. However, KCC2 levels in SNr were normalized at 3 days after TAA treatment (*P*>0.05). Protein levels of NKCC1 in the SNr at the different time points examined remained unaffected after TAA treatment (Figure 5C and D) compared to the sham controls. Similar features were observed when C57Bl/6 mice were used instead (data not shown).

**Figure 6:**
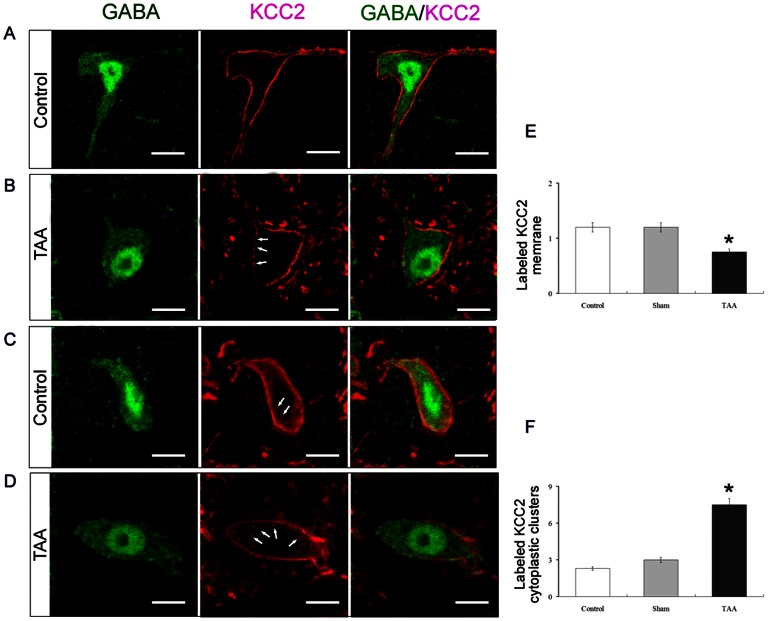
Reduced targeting of KCC2 to membrane of GABAergic neuron within the SNr following TAA treatment. (A–D) Results of immunohistochemical staining with a KCC2 - specific antibody (red) in SNr GABAergic neurons (green) of control and TAA mice. Single optical sections showing normal KCC2 staining and the discontinuous clusters of stained spots (arrows) in GABAergic neuron membranes of control (A) and TAA (B) mice. Single optical sections showing the density of cytoplasmic KCC2 - immunolabeled clusters (arrows) in control (C) and TAA (D) mice. (E) Quantification of the density of membrane labeling (ratios of labeled pixel surface per somatic perimeter) in 24 GABAergic neurons from three control mice and 27 GABAergic neurons from three TAA mice. (F) Number of cytoplasmic KCC2-immunolabeled clusters per 100 µm^2^ of somatic area. GABAergic neurons in both control and TAA mice. Bars in A, B, C and D = 10 µm. * in E and F *P*<0.05 vs normal controls.

We further analyzed KCC2 subcellular localization by immunohistochemistry in SNr GABAergic neurons after TAA treatment (Figure 6). KCC2 immunostaining in controls was abundant throughout the substantia nigra (data not shown). In the SNr, a dense matrix of processes (probably dendrites, as KCC2 is not expressed in axons [49]) was stained around GFP-immunoreactive cell bodies, presumably GABAergic neurons. After TAA injection, the intensity of KCC2 immunolabeling was reduced mainly in the SNr compared to other brainstem areas (data not shown). At higher magnifications, KCC2 immunolabeling in the SNr surrounded GABAergic neuron bodies, suggesting a preferential localization in GABAergic neuron plasma membranes (Figure 6). The membrane labeling appeared as a uniform band in control mice and also as cytoplasmic clusters in TAA-treated mice. The density of labeling in the cell membrane was significantly reduced in TAA-treated mice when compared to control (ratios of mean pixel intensities in neurons versus white matter: TAA, 4.29±0.13; control, 4.86±0.11; n = 204; *P*<0.05; Figure 6E). Conversely, the density of cytoplasmic clusters was higher in TAA treated mice, when compared to untreated or vehicle-treated controls (Figure 6F and not shown). These results suggest that KCC2 is displaced from the plasma membrane in GABAergic neurons after TAA treatment. Similar immunohistochemical results were observed when C57Bl/6 were used instead (not shown).

To further validate previous data the relative expression of KCC2 in the plasma membrane compared to cytoplasmic compartments was quantified by western blot in tissue obtained from the lumbar spinal cord of untreated control and TAA-treated transgenic mice (see Methods S1). TAA caused a significant reduction in the relative amount of plasmalemmal versus cytoplasmic KCC2 expression (*P*<0.05), compared to control mice (Figure S1).

In order to analyze changes in ion transporters known to be expressed in other brain areas which were previously found involved in the pathology of HE, we performed triple immunofluorescent stainings of voltage-sensitive chloride channel 2 (ClC-2), TH (detecting dopaminergic neurons in SNc) and GFP (detecting GABAergic neurons in SNr in transgenic mice used) in substantia nigra of untreated and TAA-treated transgenic mice (see Methods S2). CIC-2 immunoreactivity was found in TH-positive (dopaminergic) neurons of the SNc, but not in the GFP-positive (GABAergic) neurons of the SNr (Figure S2). Interestingly, CIC-2 immunoreactivity remained unaffected after TAA treatment in the SNc (Figure S2C).

These results were confirmed by qPCR at the mRNA level (see Methods S5, Figure S3). As shown in Figure S3, CLC-2 mRNA levels exhibited a mild increase both in the sham controls (120.5±1.6% of control) and the TAA group (110.0±2.0% of control) 1 day after treatment, but the changes had no statistical significance. Moreover, there were no significant differences in the expression of CLC-2 between TAA and vehicle-treated controls at either 2 d or 3 d. Similar results were obtained with C57Bl/6 mice (data not shown).

In order to study the specificity of changes KCC2 expression levels in the SNr, double immunofluorescence stainings of KCC2 and glycine were performed in the spinal cord of control and TAA-treated C57BL/6 mice (see Methods S3 and S4). Immunoreactivity for KCC2 (Figure S4) was found in the perikarya of the glycinergic neurons mainly at the spinal dorsal horn. It is worth noting that, after TAA injection, the intensity of KCC2 immunoreactivities in the spinal cord did not change significantly (Figure S4B).

### TAA treatment does not influence number of SNr neurons

**Figure 7:**
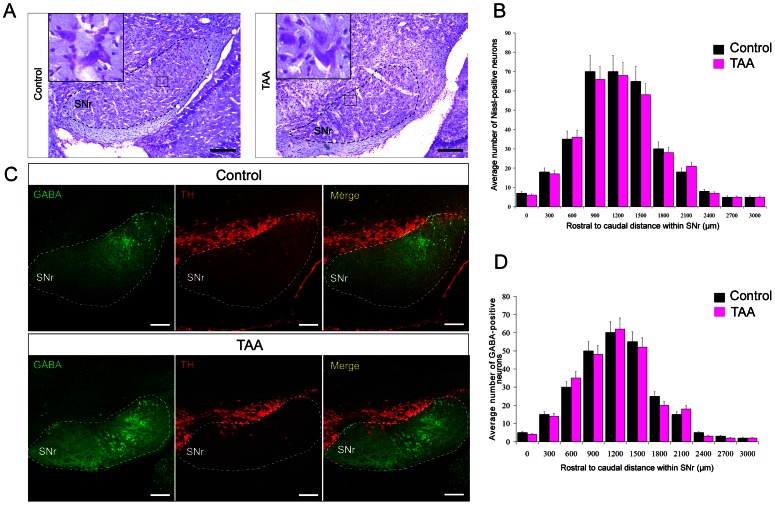
TAA did not alter the number of Nissl - positive and and GABAergic neurons within the SNr. (A) Representative Nissl staining in SNr neurons of normal control (control) mice and TAA - injected (TAA) mice. The borders of the SNr were indicated by the black dotted line. Inset showing high magnifications of selected square area of Nissl - staining section indicated there was no morphological difference between normal and TAA-injected mice. (B) Graph displaying no alteration of the distribution of Nissl - positive neurons within the SNr between control and TAA groups. (C) Immunohistochemical analysis of GABAergic neurons (green) in the SNr of normal control (control) mice and TAA - injected (TAA) mice. TH-immunohistochemistry (red) was used to determine the location and outline of the SNr of the sections, and the borders of the SNr are indicated by the white dotted line. (D) Graph displaying no changes in the distribution of GABA - immunoreactive neurons within the SNr between control and TAA groups. Bars in A and C = 100 µm.

We then analyzed whether liver acute injury may alter the number of SNr neurons in transgenic mice. Nissl staining showed that positive cells were evenly distributed throughout the SNr with no apparent regional bias among experimental groups (Figure 7A). The distributions of Nissl - positive neurons along the rostrocaudal plane of the SNr remained also unchanged with treatments and peaked at 1,000–2,000 µm from the rostral pole of the SNr at a point that corresponded with sections showing the widest diameter of the SNr (Figure 7B). Higher magnifications showed no morphological differences in between experimental groups. GFP (GABAergic neurons; SNr) and TH (dopaminergic neurons; SNc) double fluorescent stainings showed that the TAA injections did not alter numbers of GABAergic neurons in the SNr compared to control (Figure 7C). Morphological results showed that the distribution of GFP - immunoreactivity along the rostrocaudal plane of the SNr also peaked 1,000–2,000 µm from the rostral pole of the SNr at a point that corresponded to sections having the widest diameter of the SNr (Figure 7D). These results suggested that the number of the GABAergic neurons within the SNr remained unaffected after TAA treatment. Similar results were observed when these experiments were performed with C57Bl/6 mice (not shown).

### Effect of intranigral injection of drugs

**Figure 8:**
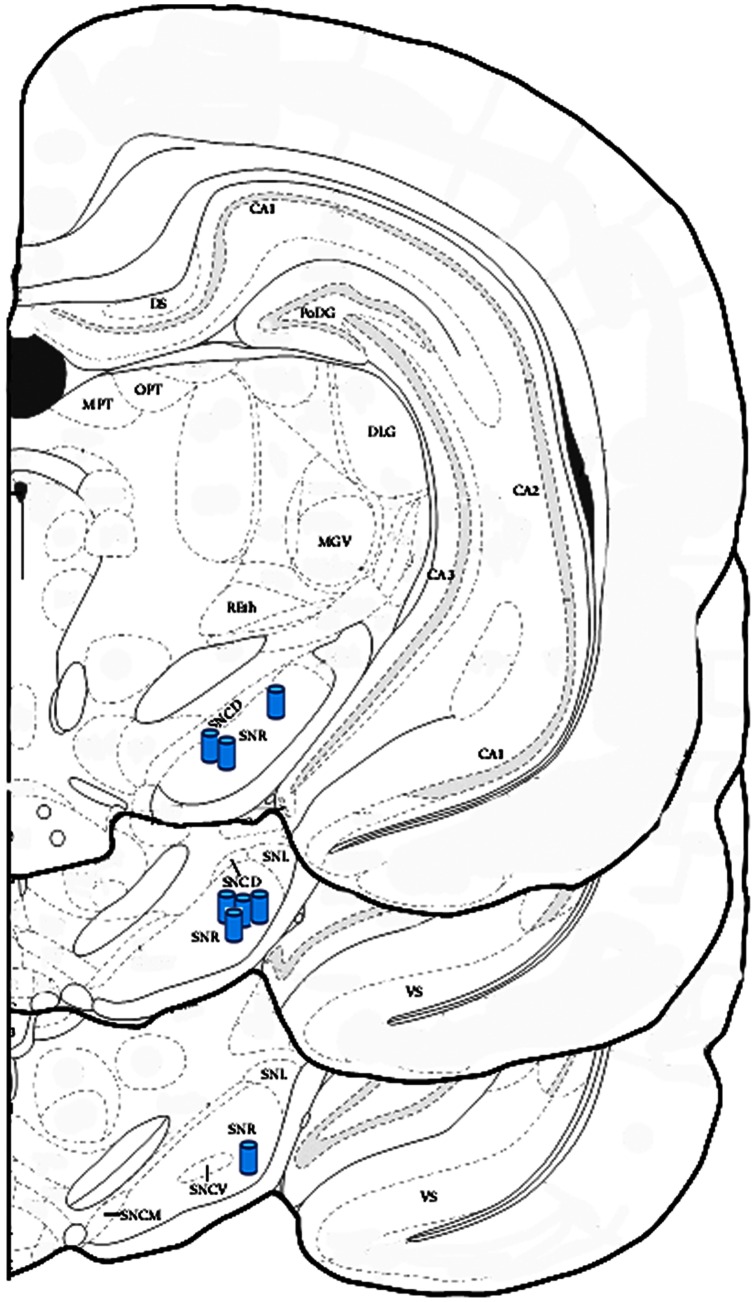
Schematic drawing placement of injectors for the mice receiving drugs (blue cylinders) in the left substantia nigra.

As illustrated in Figure 8, the tip of the syringe needle was positioned in the central part of the substantia nigra and did not damage adjacent structures. The effect of intranigral blockage of KCC2 or NKCC1 on the motor activity of transgenic mice was evaluated by an open field test. In addition, precise intranigral delivery of drugs was proven by application of a dye and subsequent sectioning (Figure S5).

**Figure 9:**
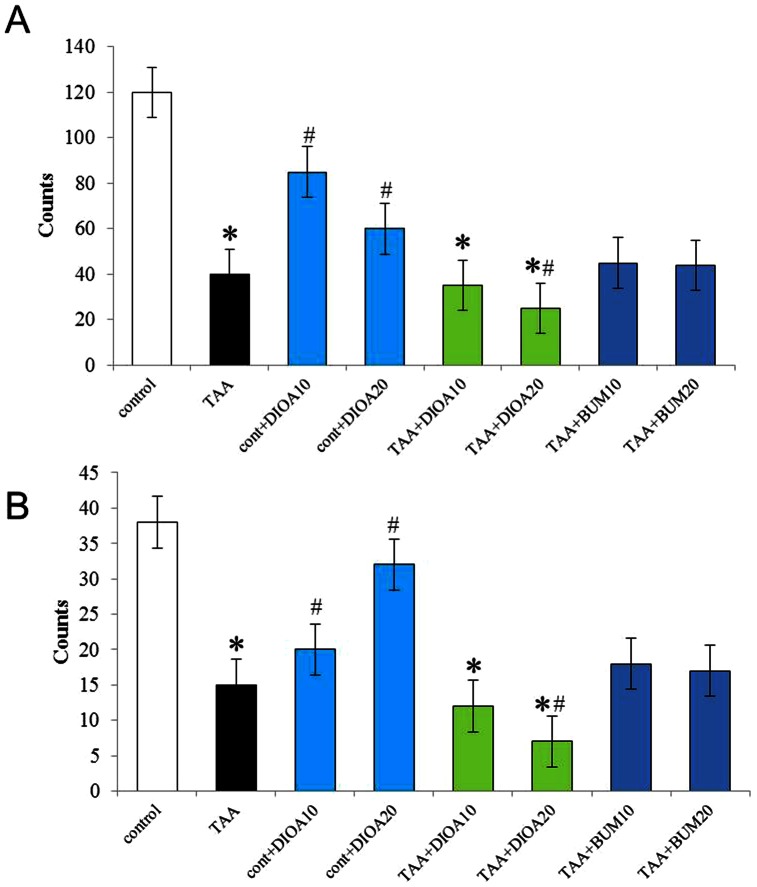
Effect of intranigral blockage of KCC2 or NKCC1 on the motor activities in GAD67 - GFP knock - in mice of each group, evaluated by open field test. Horizontal (A) and vertical (B) locomotor activity was measured. TAA induced significant hypolocomotion. Blockage of KCC2 by intranigral injection of DIOA (a KCC2 blocker) at two concentrations (10 and 20 µg/0.5 µl) induced motor function impairment in both horizontal and vertical activities in the normal mice (cont+DIOA10 and cont+DIOA20), and higher concentration of DIOA (20 µg/0.5 µl) had much more remarkable motor impairment. In addition, blockage of KCC2 by intranigral injection of DIOA (with higher concentration, 20 µg/0.5 µl) deteriorated hypolocomotion in both horizontal and vertical activities in TAA mice (TAA+DIOA20), although a lower concentration of DIOA (10 µg/0.5 µl) had no such effect (TAA+DIOA10) on TAA-induced hypolocomotion. Blockage of NKCC1 by intranigral injection of bumetanide (BUM) (a selective inhibitor of NKCC1) with two concentrations (10 and 20 nmol/0.5 µl) had no effect on TAA-induced hypolocomotion in both horizontal and vertical activities (TAA+BUM10 and TAA+BUM20). * *P*<0.05 vs. control and # *P*<0.05 vs TAA - treated mice.

As shown in Figure 9, blockage of KCC2 by intranigral injection of DIOA (a KCC2 blocker) at two concentrations (10 and 20 µg/0.5 µl) induced motor function impairment in both horizontal (Figure 9A) and vertical (Figure 9B) activities in the untreated mice. Higher concentrations of DIOA (20 µg/0.5 µl) had much more remarkable motor impairment. In addition, blockage of KCC2 by intranigral injection of DIOA (with higher concentration, 20 µg/0.5 µl) further deteriorated hypolocomotion in both horizontal and vertical activities in TAA-treated mice, although the lowest concentration of DIOA used (10 µg/0.5 µl) had no such effect.

Moreover, blockage of NKCC1 by intranigral injection of bumetanide (BUM) (a selective inhibitor of NKCC1) with two concentrations (10 and 20 nmol/0.5 µl) had no effect on TAA-induced hypolocomotion in both horizontal and vertical activities. No significant differences in motor functions were observed in between untreated and vehicle-treated controls (*P*>0.1 in each group) and a similar effect of intranigral injection of drugs was observed in C57Bl/6 mice (data not shown). At the same time, intranigral treatment with BUM led to similar liver architecture as that of TAA-injected mice (Figures 1C and c).

**Figure 10:**
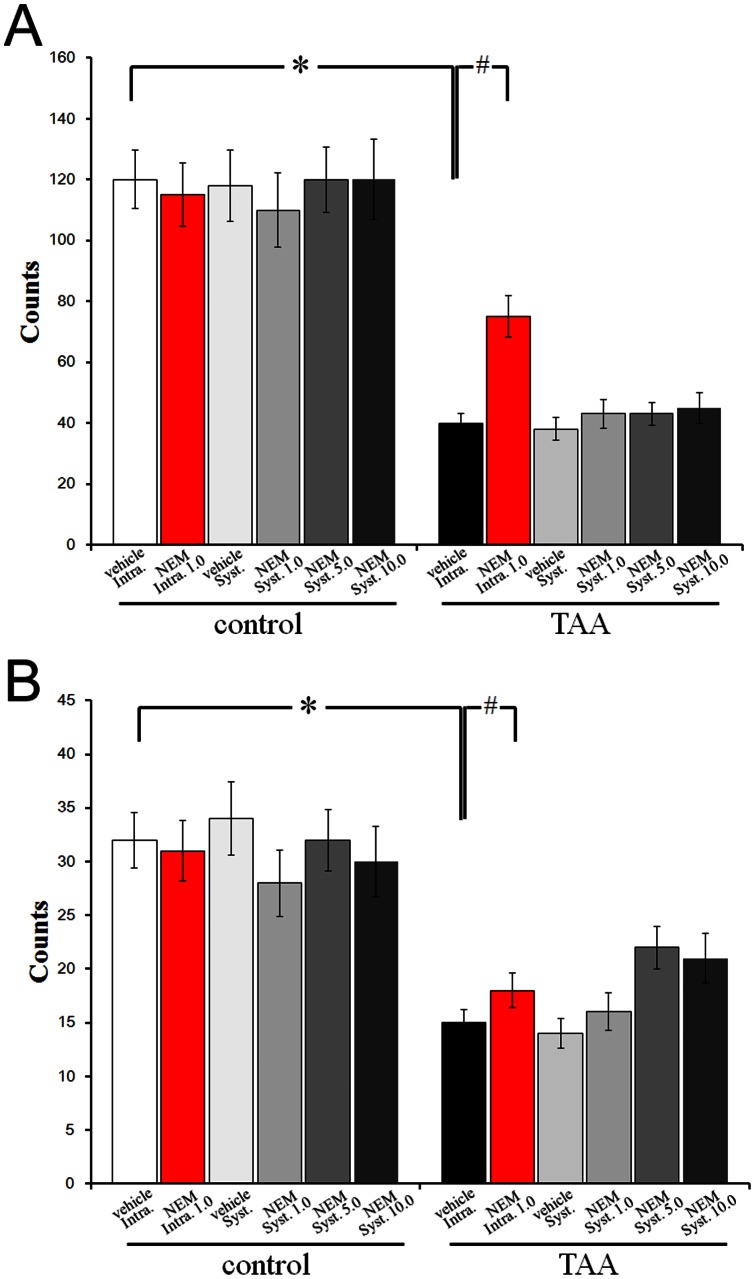
Effect of systemic injection (intraperitoneally) or intranigral activation of KCC2 with NEM on the motor activities in GAD67 - GFP knock - in mice, evaluated by open field test. Horizontal (A) and vertical (B) locomotor activity was measured. TAA induced significant hypolocomotion. Activation of KCC2 by intranigral injection of NEM (a KCC2 activator) at the concentration of 1 mmol/0.5 µl ameliorated motor function impairment in both horizontal and vertical activities in TAA-treated mice (TAA+NEM Intra. 1.0). However, activation of KCC2 by systemic injection of NEM with one of the three concentrations (1.0 mmol/0.5 µl, 5.0 mmol/0.5 µl, and 10.0 mmol/0.5 µl) or vehicle DMSO had no effect on motor function impairment in both horizontal and vertical activities in TAA mice (TAA +NEM Syst. 1.0; TAA +NEM Syst. 5.0; TAA; +NEM Syst. 10.0; vehicle Syst.). Both intranigral and systemic activation of KCC2 had no effect on TAA-induced hypolocomotion in both horizontal and vertical activities (control). * *P*<0.05 vs. control and # *P*<0.05 vs TAA - treated mice.

To further confirm previous results we have then intranigrally injected *N*-ethylmaleimide (NEM, a KCC2 activator) at the concentration of 1 mmol/0.5 µl. As shown in Figure 10, this treatment was able to ameliorate motor function impairment in both horizontal (Figure 10A) and vertical (Figure 10B) activities in TAA-treated mice. When NEM was intraperitoneally applied at different concentrations (1.0, 5.0 or 10.0 mM) and DMSO as vehicle, no effects on motor function impairment were observed in TAA-treated mice (Figure 10). No effects on motor function activities were found in untreated mice. Similar results were obtained in C57Bl/6 mice (data not shown).

Histopathologic examination of liver specimens indicated that the livers of untreated mice with intranigral treatment of DIOA (20 µg/0.5 µl) looked unaltered (data not shown). Additionally, as shown in Figure 1, HE staining revealed that intranigral treatment with bumetanide (20 nmol/0.5 µl) could not ameliorate necrosis and inflammation in the livers of mice treated with TAA. None of the intranigral treatments alone was able to modify levels of serum transaminases in either untreated or TAA-treated mice (not shown).

The levels of both blood and brain ammonia in TAA plus DIOA or bumetanide were not statistically different from those of TAA-treated animals (Table S1). Moreover, water content in TAA plus DIOA or bumetanide remained unaffected after TAA treatment (Table S1). Similar effects were found after intranigral application of same drugs in C57Bl/6 mice (data not shown).

To further validate the HE model and described the effects of local application of different drugs, releases of GABA and glutamate were measured at cerebral cortex and SNr of the brain using microdialysis (see Methods S6 and S7). Previous studies and our current data confirm that acute hepatic failure induced by TAA treatment can increase GABA [39] and glutamate levels [5] in the cerebral cortex. TAA induced an increase in both GABA (Figure S6A) and glutamate (Figure S6B) release compared with those of controls, causing a maximal 44% and 245% increase (*P*<0.05), respectively. Intranigral injection of NEM had no effect on cerebral cortex GABA release (Figure S6A) throughout the whole observation period. However, during the first 45 min after intranigral injection of NEM (a KCC2 activator), significantly reduced glutamate concentration levels were observed in the cerebral cortex when compared to intranigrally vehicle injected TAA-treated mice (*P*<0.05; Figure S6B). Interestingly, both GABA (Figure S6C) and glutamate (Figure S6D) levels when measured at the SNr remained unaffected after TAA treatment; nevertheless, GABA levels were risen 30 min after NEM administration and reached maximal values 90 min later (Figure S6C). DIOA (a KCC2 blocker) had no effect on GABA release within the SNr during the whole observation period (90 minutes) (Figure S6C). Either DIOA or NEM had no effect on glutamate release within the SNr (Figure S6D). Similar effect of intranigral injection of drugs was observed in untreated mice (data not shown).

## Discussion

In the present study, we provide evidences involving chloride homeostasis in the hypolocomotor phenotype associated with HE specifically in GABAergic neurons of the SNr. Among them, chloride imaging analyses showed an increase in intracellular chlroride concentration within this neuronal population after TAA treatment. Regarding mechanisms mediating this feature, both mRNA and protein KCC2, a chloride extruder, were downregulated in this acute hepatotoxic model within the SNr; in contrast, expression levels of the chloride intruder NKCC1 remained unchanged. In addition, its expression in GABAergic neuronal cell membrane was reduced. Blockade of KCC2 by intranigral application of DIOA in control group animals resulted in a reduction in locomotor activity, and it deteriorated the hypolocomotor phenotype in TAA-treated mice. Furthermore, activation of KCC2 by intranigral injection of NEM was found to ameliorate motor function impairment in both horizontal and vertical activities in TAA-treated animals.

Interestingly, microdialysis studies with/out intranigral application of specific KCC2 bockade/activator drugs, measuring GABA and glutamate levels in the cerebral cortex, were consistent with previous results. In this study, GAD67-GFP knock-in mice were used as tools for studying both the physiological and anatomical profiles of GABAergic neurons [26], [40]–[42]. It is generally established that controlled expression of GFP in neurons of transgenic mice is biologically inert [26]. Prolonged expression of GFP had no detectable effect on synaptic structure [43]. No apparent effect on cellular physiology [44] or behavior [45] of transgene expression was found in GABAergic neurons of the SNr. Hence, it is not surprising that in the present study the GAD67-GFP knock-in mice with TAA administration had characteristics of development of encephalopathy and hypolocomotion indistinguishable from non-transgenic mice.

Jayakumar et al. have previously involved an upregulation of NKCC1 expression in the mediation of astrocyte swelling in vitro [44], [45], and intraperitoneal injection of the NKCC1 inhibitor bumetanide was found to attenuate TAA-induced brain edema [36]. In this study, a downregulation in KCC2 expression within the SNr was found involved in the locomotor phenotype of an acute liver toxicity model, which therefore results in an increase in intracellular chloride. While Jayakumar and collaborators were able to block NKCC1 by intraperitoneal injection of specific drugs, we found that a local intranigral drug application was required to obtain efficiency in treatments. These findings highlight that abnormal chloride homeostasis is indeed involved in locomotor phenotype of a brain disorder induced by acute liver failure. Hence, drugs aimed at restoring normal chloride homeostasis could be a potential approach to treat hepatic failure in order to prevent locomotor deficiencies associated with HE.

Chloride is the most abundant anion in metazoans [19], [46]. Its diffusion through membrane anion channels enables several life-supporting processes, such as volume regulation, trans-epithelial salt transport, fluid secretion, acidification of inner and outer cell compartments and electrical excitability. Until the 1980s, the predominant paradigm was that chloride would remain in equilibrium across most cell membranes thus preventing alterations in physiological processes. It is now broadly accepted that active chloride transport is a common feature in cell physiology, maintaining the electrochemical equilibrium and contributing to neural signaling [19], [46]. Moreover, it is also well established that chloride transporters play essential roles in active regulation of intracellular chloride concentration for proper neuronal function. Abnormal chloride homeostasis caused by upregulation of NKCC1, downregulation of KCC2 or concurrency of both features have been previously reported in different anatomical neural structures such as hippocampus, spinal cord and cortex, and it was involved in the pathophysiology of other neurological disorders [23]–[25].

In the present study, both KCC2 mRNA and protein expression levels were found downregulated in the SNr of TAA-treated mice. Interestingly, at 3 days after TAA application while KCC2 mRNA levels were kept downregulated its protein expression was found normalized. The exact reason for this discrepancy between RNA and protein levels remains to be addressed.

Blockade of either KCC2 (with DIOA) or NKCC1 (with bumetanide) in the SN was not able to influence the hepatic function in control or TAA-treated groups. These results are consistent with i.e. the absence of KCC2 expression in the liver [47]. Moreover, our data related to the application of bumetanide was partially consistent with results reported by Jayakumar et al [36] showing no effect on brain water content after TAA treatment. However, they have also shown that 20 mg/kg bumetanide displayed a therapeutic effect in reducing cell swelling and brain edema in TAA-treated animals. This discrepancy might be due to differences in experimental design. While we injected this drug intranigrally, Jayakumar and his collaborators applied it intraperitoneally. Interestingly, our results showed that intranigral application of bumetanide could ameliorate the induction of LDH serum levels caused by TAA, which is consistent with previous findings [48].

The activation of KCC2 with NEM was found to ameliorate motor function impairment in TAA-treated mice, suggesting a possible beneficial effect of NEM on HE.

In the substantia nigra, relatively little data were available regarding the mechanisms mediating chloride regulation in dopaminergic neurons of the SNc and nondopaminergic neurons (the majority of them being GABAergic) of the SNr [49], [50]. Gulácsi et al. have involved KCC2 in the maintenance of the low intracellular Cl^−^ concentration in nigral GABAergic neurons of rats [49]. Interestingly, Galanopoulou et al. have found sex differences in the expression of KCC2 mRNA in both anterior SNr neurons, which are exclusively GABAergic, and posterior SNr neurons, which are predominantly GABAergic [50].

In order to analyze changes in ion transporters expressed in other brain areas involved in the pathology of HE, we have performed triple immunofluorescent stainings of voltage-sensitive chloride channel 2 (ClC-2), TH (detecting dopaminergic neurons in SNc) and GFP (detecting GABAergic neurons in SNr) in substantia nigra of normal and TAA-treated transgenic mice. Real-time RT-PCR have been performed to detect the relative mRNA levels of CIC-2 within the SNr with or without TAA treatment. Our results suggest that CIC-2 is not likely involved in the acute form of HE induced by TAA.

In addition, in order to study the specificity of changes in KCC2 expression in SNr, we have performed double immunofluorescent stainings of KCC2 and glycine in spinal cord of control and TAA-induced hepatotoxic transgenic mice. Our data suggest that other inhibitory neurons, such as the glycinergic neurons of the spinal, are likely not affected in the pathology of HE.

One may argue that the observed locomotor amelioration is independent of liver damage. Numerous substances have been found to influence liver functions and the development and severity of HE [1], [2]. In example, simvastatin (the 3-hydroxy-methyl-3-glutamyl coenzyme A reductase inhibitor) [51] and chronic thyroid hormone inhibition by methimazole [52] treatments were shown to improve motor activity and ameliorate encephalopathy in TAA- or bile-duct ligation induced HE models, respectively. At the same time, these compounds significantly ameliorate some liver biochemistry parameters (including AST and ALT transaminases and ammonia). However, our results are more in line with a recent report showing that chronic administration of indomethacin, a well-known inhibitor of cyclooxygenase isoenzymes, could prevent the impairment of locomotor activity in rats with portal vein ligation (PVL) – induced HE, but not PVL-induced hyperammonemia [53]. These findings raise the intriguing possibility that in animals with acute liver injury and HE, specific neurotransmission in brain, rather than liver function parameters, might be shown to correlate with motor activities. Overall, therapies that target chloride homeostasis may contribute to the amelioration of HE, but further clarification is required.

The main tenet of all theories of the pathogenesis of HE is firmly accepted: nitrogenous substances derived from the gut adversely affect brain function. Abnormalities in glutamatergic, serotoninergic, GABAergic, and catecholamine pathways, have been described in experimental HE [54]. In general, the new data are consistent with the progression of HE to coma being associated with increasing efficiency of the inhibitory neurotransmitter systems (GABA - benzodiapine, serotonin) and depression of the function of the excitatory glutamatergic system. The current therapy for HE is still limited to traditional nonspecific approaches including correction of identifiable precipitating factors, reduction of intestinal absorption nitrogenous substances and reduction of portal-systemic shunting. Despite much work over many years to refine these traditional approaches, in practice, they are usually associated with only partial and inconsistent improvements in the severity of HE. Future progress in therapy of HE is likely to include pharmacological intervention aimed at correcting or restoring altered chloride homeostasis.

## Conclusions

Our data suggest that altered chloride homeostasis likely mediates the pathophysiology of hypolocomotion in HE. Drugs aimed at restoring normal chloride homeostasis would be a potential treatment for encephalopathies associated with hepatic failure.

## Supporting Information

Table S1 **The levels of blood ammonia and brain ammonia, as well as the water content in different experimental groups.** TAA treatment induced significant increases in the levels of blood ammonia and brain ammonia, as well as the water content. The intranigral injection of DIOA (a KCC2 blocker, 20 µg/0.5 µl), or bumetanide (BUM, a selective inhibitor of NKCC1, a 20 nmol/0.5 µl) could not alter these increases in mice treated with TAA.
(DOCX)

Figure S1 **Relative amount of KCC2 in the plasma membrane (mb) compared to cytoplasmic compartments in the lumbar spinal cord of normal control (contol) and TAA-induced hepatotoxic (TAA) transgenic mice, quantified from western blots.** TAA caused a significant reduction in the relative amount of plasmalemmal versus cytoplasmic KCC2, compared to control mice. * *P*<0.05 vs control.
(TIF)

Figure S2 **Analysis of ClC-2 expression in the SNc following TAA injection. A and B, Immunofluorescent stainings of the distribution of ClC-2-immunolabeled neurons in the substantia nigra of GAD67-GFP knock-in mice.** A, Low magnification immunofluorescent photographs showing the presence of CIC-2-immunopositive cells (red) in TH-positive (dopaminergic) neurons (blue) of the SNc, but not in GABAergic neurons (green) of the SNr. B, High-magnification immunofluorescent photographs showing that ClC-2 is mainly present in the perikarya, but not in dendrites of the SNc neurons. Scale bars: A, 100 µm; B, 25 µm. C, Histograms demonstrating the intensity of ClC-2 immunoreactivity in the SNc from normal controls, sham and TAA-treated transgenic mice. All three experimental groups showed a rather similar intensity.
(TIF)

Figure S3 **Real-time quantitative RT-PCR experiments were performed using SYBR green for analysis of ClC-2 mRNAs expression of TAA-injected mice at different time points and sham controls.** All data were normalized for levels of GAPDH expression within the same sample. Data are calculated as percentages of the average value of controls.
(TIF)

Figure S4 **Expression of ClC-2 mRNA within the SNc of substantia nigra following TAA injection.** Analysis of KCC2 changes expressed in the spinal cord following TAA injection. A and B, The distribution of KCC2- and glycine- immunolabeled neurons in the spinal cord of wild-type C57BL/6 mice. A, Low-magnification immunofluorescent photographs show the presence of KCC2- (red) and glycine- (green) immunoreactivities in the spinal cord. B, High-magnification fluorescent photographs demonstrate that KCC2 is mainly present in the perikarya of the glycinergic neurons. Scale bars: A, 100 µm; B, 25 µm. C, Histograms correspond to quantification of the intensity of KCC2 immunoreactivity in the spinal cord from normal controls, sham and TAA-treated transgenic mice. All three experimental groups showed a rather similar intensity.
(TIF)

Figure S5 **Photomicrograph showing the correct placement of intranigral injections.** Note: the arrow indicates the presence of the dye (methylene blue) in the SNr. Scale bar = 200 µm.
(TIF)

Figure S6 **Measurements of GABA (A and C) and glutamate (B and D) releases at the cerebral cortex (A and B) and SNr (C and D) of untreated (cont) and TAA-treated transgenic mice at the ipsilateral side by using a microdialysis method.** The effects of NEM and DIOA applications in untreated and TAA-treated mice on similar parameters are also shown. Data are expressed as percentage of basal pre-treatment levels (calculated as the mean of the two samples preceding the treatment) and are mean ± SEM of 4–8 animals. Basal dialysate levels of GABA and GLU were 42.0±11.8 and 268.3±25.9 nM in Cortex, and 7.0±0.4 and 53.6±5.0 nM in SNr, respectively. Statistical analysis was performed by two-way RM ANOVA followed by contrast analysis and the sequentially rejective Bonferroni's test. A, TAA increased GABA release compared with those controls, causing a maximal 44% increase (*P*<0.05). NEM had no effect on GABA release, compared with those after vehicle. B, TAA increased Glu release compared with those controls, causing a maximal 245% increase (*P*<0.05). NEM significantly decreased Glu concentrations, causing a maximal 22% reduction (*P*<0.05), compared to those after vehicle. C, GABA concentrations in SNr remained unaltered following the administration of TAA at the ipsilateral side. In addition, GABA release levels in the SNr were significantly elevated 30 min after NEM (a KCC2 activator) administration and reached maximal values 120 min after NEM administration. However, DIOA (a KCC2 blocker) had no effect on GABA release within the SNr during the whole observation time (90 min). D, Glu concentrations at the ipsilateral SNr were not altered by TAA. Either DIOA or NEM had no effect on Glu release within the SNr during the whole observation time (90 min), in both groups. * *P*<0.05 *vs* the groups of mice without TAA; # *P*<0.05 *vs* the groups of mice received vehicle with TAA.
(JPG)

Methods S1 **Supplementary methods.**
(DOC)
