# Supplementary material for: Abnormal Chloride Homeostasis in the Substancia Nigra Pars Reticulata Contributes to Locomotor Deficiency in a Model of Acute Liver Injury
Source: PLoS One. 2013 May 31;8(5):e65194. doi: 10.1371/journal.pone.0065194 (PMC3669273; doi:10.1371/journal.pone.0065194)
Supplement: Table S1 — The levels of blood ammonia and brain ammonia, as well as the water content in different experimental groups. TAA treatment induced significant increases in the levels of blood ammonia and brain ammonia, as well as the water content. The intranigral injection of DIOA (a KCC2 blocker, 20 µg/0.5 µl), or bumetanide (BUM, a selective inhibitor of NKCC1, a 20 nmol/0.5 µl) could not alter these increases in mice treated with TAA. (DOCX) [file pone.0065194.s001.docx]

Table S1

The levels of blood ammonia and brain ammonia, as well as the water content in different experimental groups. TAA treatment induced significant increases in the levels of blood ammonia and brain ammonia, as well as the water content. The intranigral injection of DIOA (a KCC2 blocker, 20 μg/0.5 μl), or bumetanide (BUM, a selective inhibitor of NKCC1, a 20 nmol/0.5 μl) could not alter these increases in mice treated with TAA.

|  | Blood ammonia  (μmol/L) | Brain ammonia  (μmol/0.2 g tissue) | Water content  (%) |
| --- | --- | --- | --- |
| Control | 100.5 ± 15.2 | 352.8 ± 25.5 | 80.6 ± 1.4 |
| Sham | 110.0 ± 15.0 | 375.2 ± 18.6 | 80.8 ± 1.1 |
| TAA | 457.4 ± 29.1 * | 1180.3 ± 39.6 * | 82.5 ± 1.2 * |
| TAA + DIOA | 495.6 ± 24.7 * | 1207.7 ± 30.4 * | 82.8 ± 1.5 * |
| TAA + BUM | 477.3 ± 20.0 * | 1144.4 ± 34.9 * | 82.3 ± 1.3 * |

* *p* < 0.05 as compared to normal controls.
