# Supplementary figures and images for: Abnormal Chloride Homeostasis in the Substancia Nigra Pars Reticulata Contributes to Locomotor Deficiency in a Model of Acute Liver Injury
Source: PLoS One. 2013 May 31;8(5):e65194. doi: 10.1371/journal.pone.0065194 (PMC3669273; doi:10.1371/journal.pone.0065194)

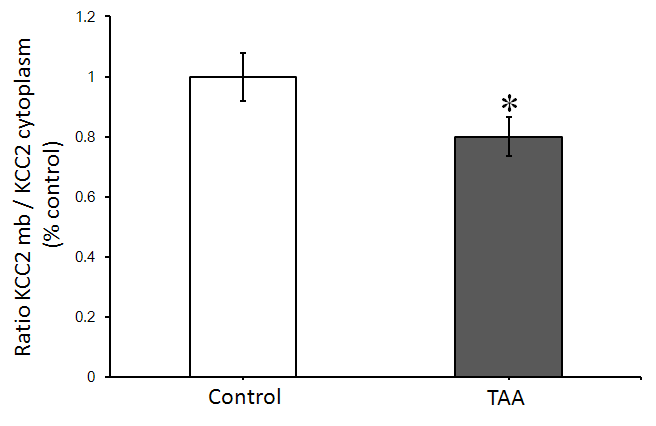

Supplement: Figure S1 — Relative amount of KCC2 in the plasma membrane (mb) compared to cytoplasmic compartments in the lumbar spinal cord of normal control (contol) and TAA-induced hepatotoxic (TAA) transgenic mice, quantified from western blots. TAA caused a significant reduction in the relative amount of plasmalemmal versus cytoplasmic KCC2, compared to control mice. * P<0.05 vs control. (TIF) [file pone.0065194.s002.tif]

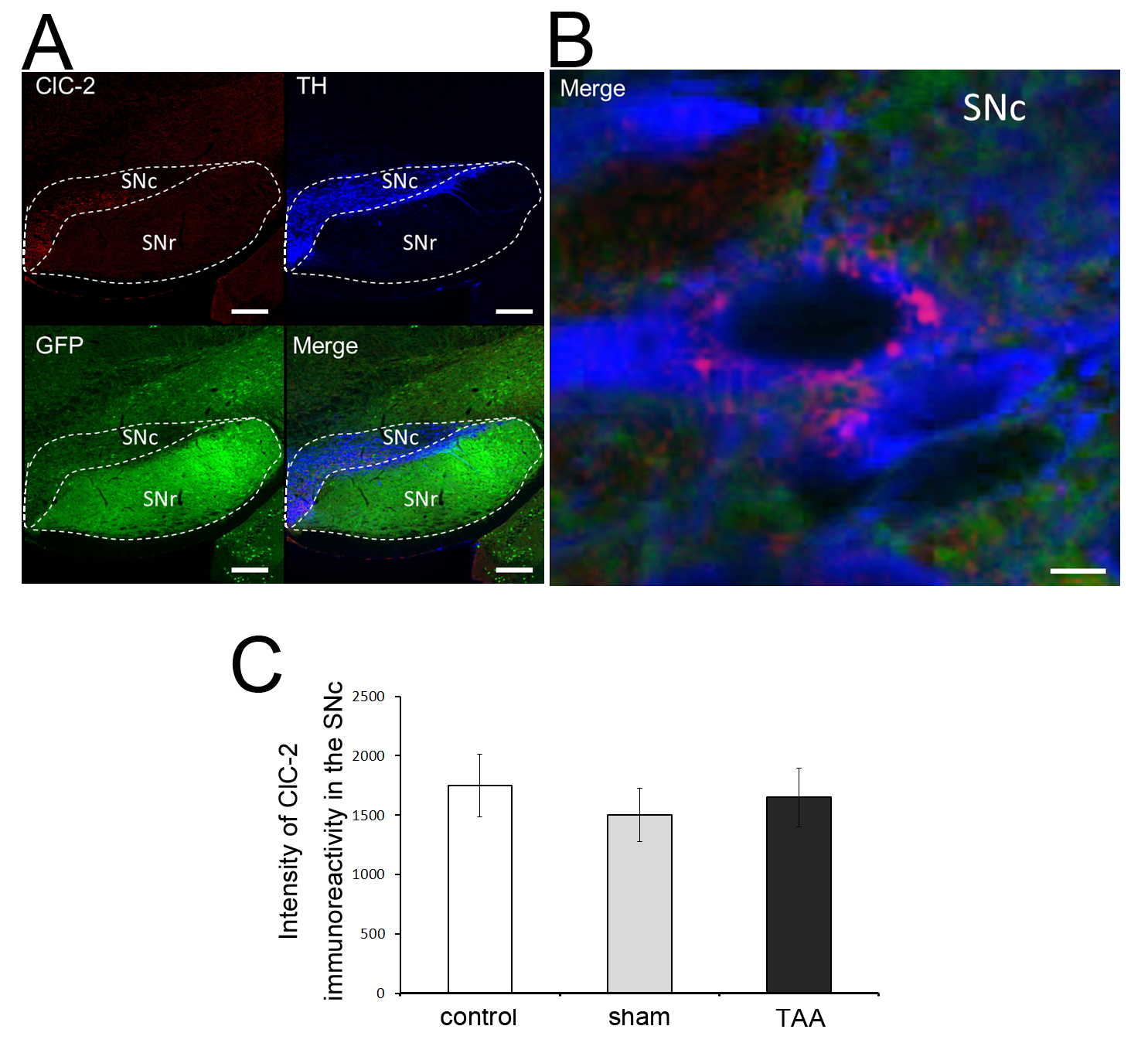

Supplement: Figure S2 — Analysis of ClC-2 expression in the SNc following TAA injection. A and B, Immunofluorescent stainings of the distribution of ClC-2-immunolabeled neurons in the substantia nigra of GAD67-GFP knock-in mice. A, Low magnification immunofluorescent photographs showing the presence of CIC-2-immunopositive cells (red) in TH-positive (dopaminergic) neurons (blue) of the SNc, but not in GABAergic neurons (green) of the SNr. B, High-magnification immunofluorescent photographs showing that ClC-2 is mainly present in the perikarya, but not in dendrites of the SNc neurons. Scale bars: A, 100 µm; B, 25 µm. C, Histograms demonstrating the intensity of ClC-2 immunoreactivity in the SNc from normal controls, sham and TAA-treated transgenic mice. All three experimental groups showed a rather similar intensity. (TIF) [file pone.0065194.s003.tif]

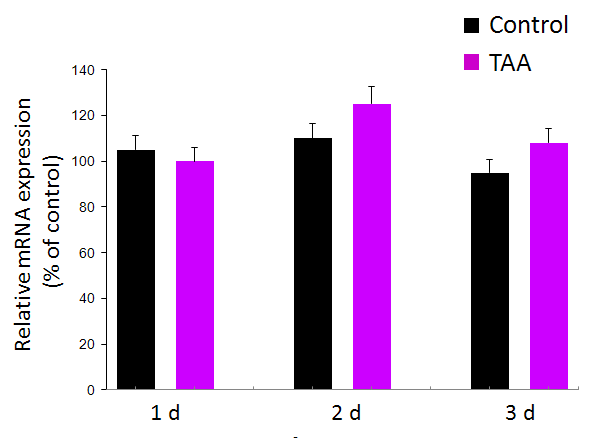

Supplement: Figure S3 — Real-time quantitative RT-PCR experiments were performed using SYBR green for analysis of ClC-2 mRNAs expression of TAA-injected mice at different time points and sham controls. All data were normalized for levels of GAPDH expression within the same sample. Data are calculated as percentages of the average value of controls. (TIF) [file pone.0065194.s004.tif]

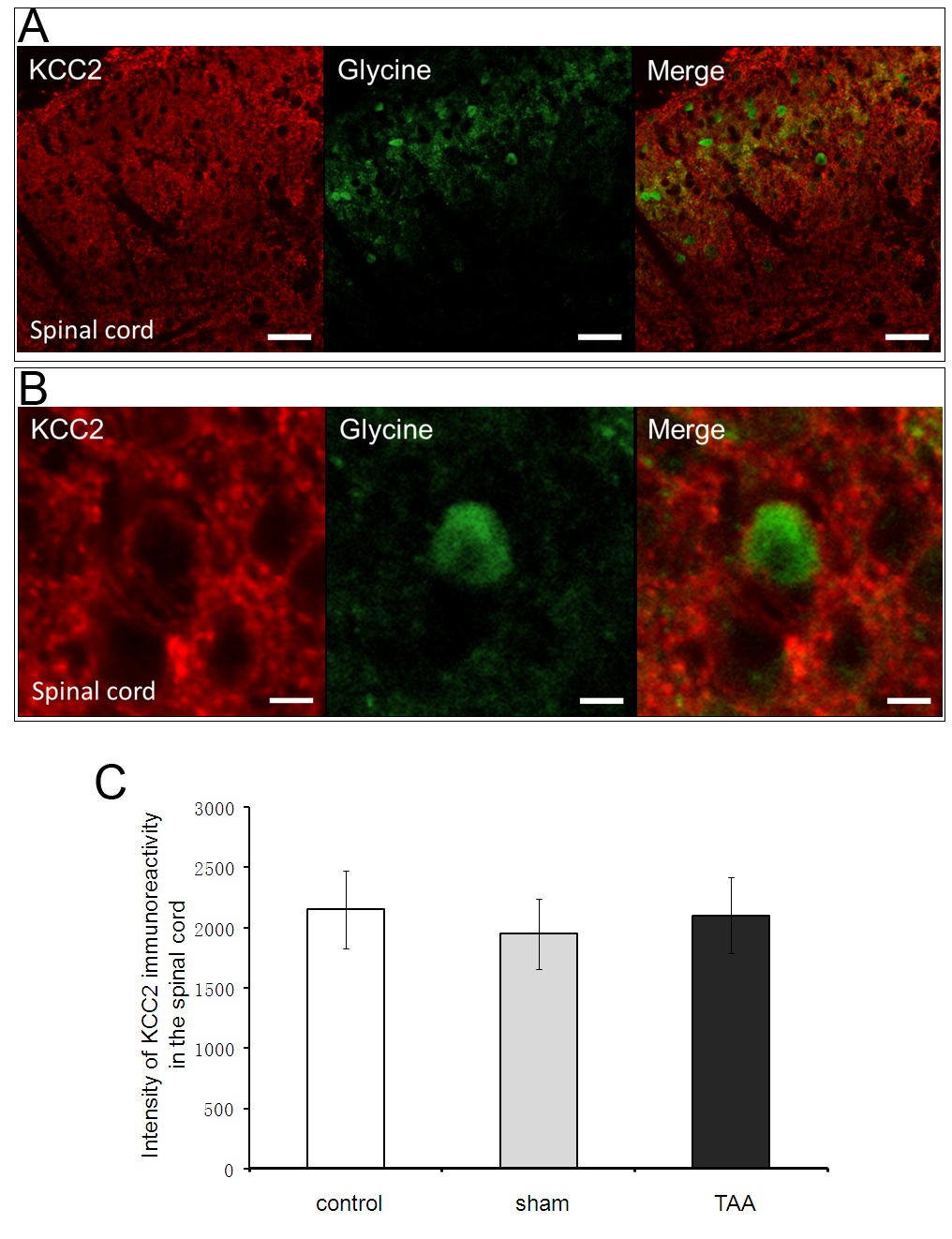

Supplement: Figure S4 — Expression of ClC-2 mRNA within the SNc of substantia nigra following TAA injection. Analysis of KCC2 changes expressed in the spinal cord following TAA injection. A and B, The distribution of KCC2- and glycine- immunolabeled neurons in the spinal cord of wild-type C57BL/6 mice. A, Low-magnification immunofluorescent photographs show the presence of KCC2- (red) and glycine- (green) immunoreactivities in the spinal cord. B, High-magnification fluorescent photographs demonstrate that KCC2 is mainly present in the perikarya of the glycinergic neurons. Scale bars: A, 100 µm; B, 25 µm. C, Histograms correspond to quantification of the intensity of KCC2 immunoreactivity in the spinal cord from normal controls, sham and TAA-treated transgenic mice. All three experimental groups showed a rather similar intensity. (TIF) [file pone.0065194.s005.tif]

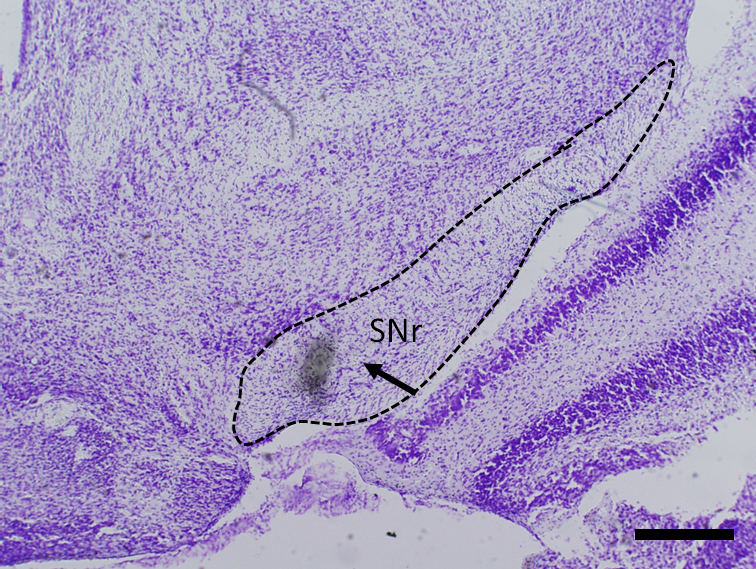

Supplement: Figure S5 — Photomicrograph showing the correct placement of intranigral injections. Note: the arrow indicates the presence of the dye (methylene blue) in the SNr. Scale bar = 200 µm. (TIF) [file pone.0065194.s006.tif]

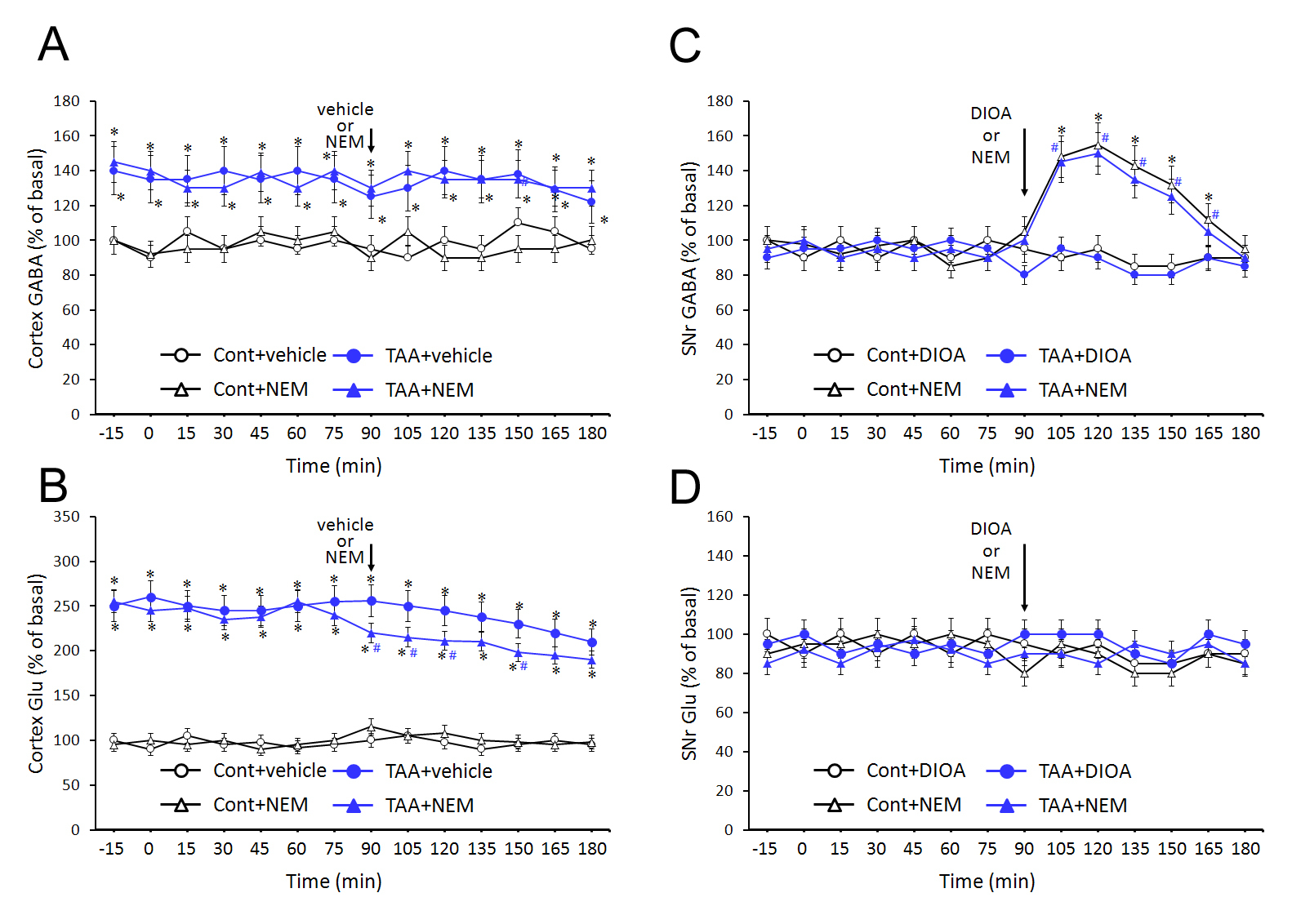

Supplement: Figure S6 — Measurements of GABA (A and C) and glutamate (B and D) releases at the cerebral cortex (A and B) and SNr (C and D) of untreated (cont) and TAA-treated transgenic mice at the ipsilateral side by using a microdialysis method. The effects of NEM and DIOA applications in untreated and TAA-treated mice on similar parameters are also shown. Data are expressed as percentage of basal pre-treatment levels (calculated as the mean of the two samples preceding the treatment) and are mean ± SEM of 4–8 animals. Basal dialysate levels of GABA and GLU were 42.0±11.8 and 268.3±25.9 nM in Cortex, and 7.0±0.4 and 53.6±5.0 nM in SNr, respectively. Statistical analysis was performed by two-way RM ANOVA followed by contrast analysis and the sequentially rejective Bonferroni's test. A, TAA increased GABA release compared with those controls, causing a maximal 44% increase (P<0.05). NEM had no effect on GABA release, compared with those after vehicle. B, TAA increased Glu release compared with those controls, causing a maximal 245% increase (P<0.05). NEM significantly decreased Glu concentrations, causing a maximal 22% reduction (P<0.05), compared to those after vehicle. C, GABA concentrations in SNr remained unaltered following the administration of TAA at the ipsilateral side. In addition, GABA release levels in the SNr were significantly elevated 30 min after NEM (a KCC2 activator) administration and reached maximal values 120 min after NEM administration. However, DIOA (a KCC2 blocker) had no effect on GABA release within the SNr during the whole observation time (90 min). D, Glu concentrations at the ipsilateral SNr were not altered by TAA. Either DIOA or NEM had no effect on Glu release within the SNr during the whole observation time (90 min), in both groups. * P<0.05 vs the groups of mice without TAA; # P<0.05 vs the groups of mice received vehicle with TAA. (JPG) [file pone.0065194.s007.jpg]
