# Supplementary material for: Abnormal Chloride Homeostasis in the Substancia Nigra Pars Reticulata Contributes to Locomotor Deficiency in a Model of Acute Liver Injury
Source: PLoS One. 2013 May 31;8(5):e65194. doi: 10.1371/journal.pone.0065194 (PMC3669273; doi:10.1371/journal.pone.0065194)
Supplement: Methods S1 — Supplementary methods. (DOC) [file pone.0065194.s008.doc]

**Supplementary Methods**

**Methods S1**

**Western blots based on the separation of cytoplasmic and membrane fractions**

The SNr samples were homogenized in cold buffer and centrifuged samples at 18,000 g for 30 min at 4°C. Supernatant protein concentrations were determined. For cytoplasmic and membrane fractions, samples were homogenized in cold lysis buffer [Sucrose 320 mM in Tris-HCl pH 7.5, 10 mM sodium vanadate, 10 mM sodium fluoride, 10 mM sodium pyrophosphate, 10 mM iodoacetamide, and a cocktail of protease inhibitors (CompleteMini, Roche diagnostic)] without detergent and centrifuged them first at 7,000 g for 5 min and then at 29,700 g for 1.5 h at 4°C. The pellets were collected in lysis buffer (1% Igepal CA-630, 0.25% SDS, 1% sodium deoxycholate, 2 mM EDTA, 1 mM DTT, 10 mM sodium vanadate, 10 mM sodium fluoride, 10 mM sodium pyrophosphate, and a cocktail of protease inhibitors) without DTT (membrane-enriched fractions) and supernatants (cytoplasmic fractions). Then separated samples of KCC2 were immunoprecipitated in 7% SDS-PAGE and were transferred to a polyvinylidene fluoride membrane. After blockade in Tris-buffered saline plus 5% nonfat dry milk, membranes were exposed overnight at 4 °C to a polyclonal rabbit KCC2-specific antibody diluted 1 in 500 (Millipore) in the blocking solution. An ImmunoPure goat horseradish peroxidase–conjugated rabbit-specific antibody (1 in 500 in blocking solution, 1 h at 22 °C) for chemiluminescent detection (Pierce Biotech) was used. Signal intensities were measured with the image analysis software Quantity-One (Bio-Rad).

**Methods S2**

**Triple immunofluorescence labeling in the SNr**

The mice were deeply anesthetized with chloral hydrate and perfused transcardially with 0.1 M phosphate buffer (pH 7.4) containing 4% paraformaldehyde. The brains were removed, postfixed with the same fixative for 4 h, then placed in 0.05 M diethylpyrocarbonate-treated PBS (pH 7.4) containing 30% sucrose (w/v) overnight at 4ºC. Sagittal sections (40 μm) throughout the entire SNr were cut on a cryostat. The sections, which were fixed as described above, were incubated overnight in PBS with a mixture of 1 μg/ml guinea pig anti - GFP antibody (Sigma), rabbit anti - ClC-2 antibody (Sigma) and mouse anti - TH antibody (1 / 200, BD Biosciences Pharmingen). After a rinse with PBS, the sections were incubated for 3 h in PBS with 4 μg / ml Alexa 488 - conjugated goat anti - guinea pig IgG antibody, 4 μg / ml Alexa 594 - conjugated donkey anti - rabbit IgG (Molecular Probes, USA) and cy5 - conjugated donkey anti – mouse IgG (Molecular Probes). The sections were mounted onto gelatinized glass slides and coverslipped with 50% (v / v) glycerol and 2.5% (w / v) triethylenediamine (anti - fading reagent) in PBS. The sections were observed under a confocal microscope (FV - 1000, Olympus, Japan) with a confocal depth of 1.0 μm, appropriate laser beams and filters for Alexa 488 (excitation: 488 nm; emission: 505 – 530 nm) and Alexa 594 (excitation: 543 nm; emission: 560 nm). Finally control experiments were performed in which the primary or secondary antibody was omitted. No labeling was observed under these conditions.

**Methods S3**

**Double immunofluorescence labeling in spinal cord**

Mice were deeply anesthetized and lumbar spinal cord were removed and postfixed. The sagittal sections (25 μm) were incubated with rabbit anti - KCC2 antibody (1 / 500, Santa Cruz) and mouse anti – glycine antibody (1 / 100, Sigma) overnight, and a mixture of Alexa 488- and Alexa 594-labeled species-specific secondary antibodies. Images were obtained with a confocal laser scanning microscope FV1000 (Olympus). And control experiments in which the primary or secondary antibody was omitted were performed. No labeling was observed under these conditions.

**Methods S4**

**Intensity analysis of alterations in ClC-2 immunoreactivities (in the SNc) or KCC2 immunoreactivities (in the spinal cord)**

In order to semi-quantitatively analyze alterations in ClC-2 (in the SNc) and KCC2 (in the spinal cord) immunoreactivity intensity with or without TAA treatment, digital photomicrographs of desired sections containing ClC-2-immunopostive or KCC2-immunopositive neurons were taken at a magnification of 10× and 20×, respectively using a Olympus epifluorescent microscope. A 10× objective was used and 2–3 sections at middle level of the SNc or spinal cord were photographed and then analyzed. As for the intensity measurement, the exposure time of each image was kept constant. The images were analyzed with MetaMorph Imaging software (Molecular Devices). These black and white images, that were pseudo-colored, encode intensity information pixel by pixel over a range (0–4095) of gray scale values. The stained intensity and density of a labeled profile was then measured using “Show Region Statistics” tool. In this assay, stronger fluorescent signals (higher averaged gray values) point to higher level of protein expression. After thresholding the pictures, values of intensity were obtained.

**Methods S5**

**Primers of ClC-2 and the amplification protocol**

The primer sequences for ClC-2 was: forward primer, 5-AGGCTTCTGTCTGCTTCCA-3; reverse primer, 5-TTCCAATGAGTCTGCCAATAC-3. Amplification product: 160 bp. The amplification protocol was 3 min at 95ºC, followed by 38 cycles of 10 s at 95ºC for denaturation and 45 s at 57ºC for annealing and extension.

**Methods S6**

**Microdialysis**

Microdialysis was used to simultaneously monitor GABA and Glutamate release in the SNr and cortex with or without intranigral injection of NEM or DIOA in TAA-induced hepatotoxic and normal transgenic freely moving mice. Surgeries and drug infusions in mice were performed in accordance with previously developed models (32). In brief, needle tip placement coordinates corresponded to those in Briefly, two microdialysis probes of concentric design were stereotaxically implanted under isoflurane anesthesia (1.5% in air) into the lesioned SNr and ipsilateral cortex (1 and 2 mm dialyzing membrane, respectively), according to the following coordinates from bregma and the dural surface (mm): SNr, AP − 3.3 mm, ML − 1.25 mm, DV− 4.6 mm; cortex, AP + 1.9 mm, ML − 0.5 mm, DV − 3.8 mm (in accordance to atlas of Slotnick and Leonard, 33). Twenty-four hours after surgery, probes were perfused with a modified Ringer solution (CaCl_2_ 1.2 mmol/L, KCl 2.7 mmol/L, NaCl 148 mmol/L and MgCl_2_ 0.85 mmol/L) at a flow rate of 2.1 μL/min. After 6 h rinsing, samples were collected every 20 min for a total of 3–4 h. At least three baseline samples were collected before intranigral administration of NEM, or DIOA, or vehicle. At the end of experiment, animals were sacrificed

**Methods S7**

**Endogenous GABA and glutamate analysis**

GABA and glutamate were measured by HPLC coupled with fluorometric detection as previously described (49). Thirty microliters of o-phthaldialdehyde / mercaptoethanol reagent were added to 30 μl aliquots of sample, and 50 μl of the mixture was automatically injected onto a 5-C18 Chromsepanalyticalcolumn (3 mm inner diameter, 10 cm length; Chrompack, Middelburg, Netherlands) perfused at a flow rate of 0.48 ml/min (Beckman 125 pump; Beckman Instruments, Fullerton, CA, USA) with a mobile phase containing 0.1M sodium acetate, 10% methanol and 2.2% tetrahydrofuran (pH 6.5). GABA and Glutamate were detected by means of a fluorescence spectrophotometer FP-2020 Plus (Jasco, Tokyo, Japan) with the excitation and the emission wave lengths set at 370 and 450 nm respectively. The limits of detection for GABA and Glutamate were ~0.5 and ~1 nM, respectively. Retention times for GABA and Glutamate were 18.0 ± 0.6 min and 3.2 ± 0.5 min respectively.
